# Retention of the RNA ends provides the molecular memory for maintaining the activation of the Csm complex

**DOI:** 10.1093/nar/gkae080

**Published:** 2024-02-10

**Authors:** Patrick Irmisch, Irmantas Mogila, Brighton Samatanga, Gintautas Tamulaitis, Ralf Seidel

**Affiliations:** Peter Debye Institute for Soft Matter Physics, University of Leipzig, Leipzig 04103, Germany; Institute of Biotechnology, Life Sciences Center, Vilnius University, Vilnius 10257, Lithuania; Peter Debye Institute for Soft Matter Physics, University of Leipzig, Leipzig 04103, Germany; Institute of Biotechnology, Life Sciences Center, Vilnius University, Vilnius 10257, Lithuania; Peter Debye Institute for Soft Matter Physics, University of Leipzig, Leipzig 04103, Germany

## Abstract

The type III CRISPR-Cas effector complex Csm functions as a molecular Swiss army knife that provides multilevel defense against foreign nucleic acids. The coordinated action of three catalytic activities of the Csm complex enables simultaneous degradation of the invader's RNA transcripts, destruction of the template DNA and synthesis of signaling molecules (cyclic oligoadenylates cA_*n*_) that activate auxiliary proteins to reinforce CRISPR-Cas defense. Here, we employed single-molecule techniques to connect the kinetics of RNA binding, dissociation, and DNA hydrolysis by the Csm complex from *Streptococcus thermophilus*. Although single-stranded RNA is cleaved rapidly (within seconds), dual-color FCS experiments and single-molecule TIRF microscopy revealed that Csm remains bound to terminal RNA cleavage products with a half-life of over 1 hour while releasing the internal RNA fragments quickly. Using a continuous fluorescent DNA degradation assay, we observed that RNA-regulated single-stranded DNase activity decreases on a similar timescale. These findings suggest that after fast target RNA cleavage the terminal RNA cleavage products stay bound within the Csm complex, keeping the Cas10 subunit activated for DNA destruction. Additionally, we demonstrate that during Cas10 activation, the complex remains capable of RNA turnover, i.e. of ongoing degradation of target RNA.

## Graphical Abstract

**Graphical Abstract:**
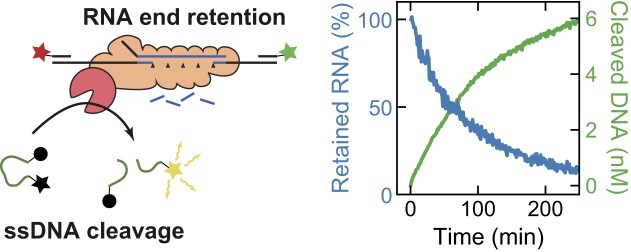

## Introduction

CRISPR (clustered regularly interspaced short palindromic repeats), together with CRISPR-associated genes (*cas*) constitute an adaptive microbial immune system which provides acquired resistance against viruses and plasmids (1). This immunity is obtained by integrating short DNA sequences (protospacers) derived from the invader genome into the host CRISPR locus. The CRISPR loci are transcribed and processed into CRISPR RNAs (crRNA) that contain a spacer flanked by parts of the repeat sequences on either side. The crRNA assembles with Cas proteins to form interference complexes that degrade foreign nucleic acids in a crRNA-guided fashion. Based on the effector complex composition, CRISPR-Cas systems are divided in two classes (2). The class 1 systems (types I, III, and IV) consist of multi-subunit effector complexes, while class 2 systems (types II, V and VI) comprise single-subunit effectors. Type I, II, V CRISPR-Cas generally target double-stranded DNA, type VI systems target RNA (1,2) and type IV systems participate in plasmid competition by recruiting the DNA helicase DinG (3).

**Figure 1.**
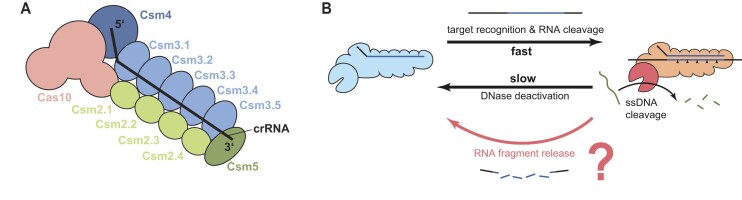
Structural scheme and RNA target-dependent ssDNA cleavage of the Csm complex. (**A**) Schematic representation depicting the subunits of the Csm complex in conjunction with the crRNA, resulting in the formation of a binary complex. (**B**) RNA target recognition by Csm leads to formation of a ternary complex that cleaves single-stranded DNA via the HD domain of the Cas10 subunit. Although rapid RNA cleavage and target recognition are observed, the DNase activity persists over an extended period. The dynamic interplay between RNA fragment release and the extent of ssDNA cleavage activity, so far, remained unresolved.

Type III CRISPR-Cas systems are of particular interest as they degrade both RNA and DNA of the invaders (2). They are divided into six subtypes (III-A to III-F), with different subunits and architectures (4). Type III CRISPR-Cas systems comprise a multi-subunit ribonucleoprotein effector complex and auxiliary effector proteins, which are recruited for defense by antiviral signaling. Type III effectors (Csm for type III-A/D/E/F, and Cmr for type III-B/C) are composed of several different Cas proteins bound to a crRNA molecule (see Figure 1A) to recognize and to target nucleic acids (5). The largest subunit of the complex, Cas10, typically contains an HD nuclease domain and two Palm (polymerase/nucleotide cyclase-like) domains. The type III CRISPR-Cas immune response is initiated by transcription of a target DNA sequence (6,7). As the RNA polymerase transcribes the foreign DNA, the nascent mRNA is recognized by base pairing with the complementary crRNA and is cleaved into single-stranded RNA (ssRNA) fragments at 6 nt intervals by the Csm3 or Cmr4 ribonucleolytic subunits (8–11). Recognition of RNA allosterically activates the Cas10 subunit. Invader-derived transcripts are distinguished from self-transcripts by testing complementarity between an 8 nt repeat-derived sequence of the crRNA 5′-tag (also termed the 5′-handle), and the corresponding 8 nt of the target RNA 3′-anti-tag (also termed the 3′-flanking sequence). Complementarity between the 5′-tag and 3′-anti-tag of an endogenous transcript blocks Cas10 activation avoiding autoimmunity. In contrast, mismatches between the 5′-tag and the 3′-anti-tag of invader transcript trigger the Cas10 activity, resulting in random single-stranded DNA (ssDNA) degradation in proximal transcription and replication bubbles by the Cas10 HD domain (6,7,12–19) and conversion of ATP into cyclic oligoadenylates (cA_*n*_, *n* = 3–6) by the Cas10 Palm domains (20–27). Both ssDNA cleavage (7,12,13,15) and cA_*n*_ synthesis (20,21,23,25) require target RNA binding but not target RNA cleavage by the effector complex. cA_*n*_ further act as secondary messengers to allosterically activate type III CRISPR-Cas-associated RNA nucleases (Csm6, Csx1) (20–24,27), DNA nucleases (Can1, Can2, Card1) (28–30), proteases (31), transcriptional regulators (32) or translational inhibitors (33) that might lead cell to dormancy or death (26,34–36).

To coordinate type III complex RNA cleavage, DNA cleavage and cA_*n*_ synthesis activities for the destruction of foreign genetic elements and for the prevention of autoimmunity, a spatiotemporal regulation mechanism was suggested (7,20,23). According to this, the target RNA cleavage by Csm3/Cmr4 RNase suppresses the Cas10 DNase and cA_*n*_ synthase activities, ensuring temporal control of DNA degradation and cA_*n*_ signaling. However, the details of how Cas10 is activated to bind and cleave ssDNA are still not clear, as ssDNA could not be visualized in structures of Csm and Cmr complex, and few conformational changes were observed in the HD domain upon RNA binding (37–39). Furthermore, it remains unclear how the activation of the type III complex by RNA binding is temporarily regulated. Previous research indicated that complex activation stops at the moment of RNA cleavage because the bound target RNA fragments dissociate (23). In contrast, it has been found that RNA cleavage occurs on a sub-minute timescale while the activation of both the DNase and the cA_*n*_ production persist over tens of minutes (7,20) (Figure 1B).

The usage of single-molecule techniques has been previously a powerful approach for resolving the recognition and the interaction dynamics of types I, II and V CRISPR-Cas effector complexes with their DNA targets (40–48). Recent single-molecule fluorescence investigations of the Csm complex from *Staphylococcus epidermidis* revealed that Cas10 is highly flexible when bound to non-self RNA target but not when bound to self-transcript (49). In this study, we applied single-molecule fluorescence experiments and bulk solution experiments to investigate the temporal regulation of RNA-activated DNA cleavage of a type III-A Csm complex from *Streptococcus thermophilus*. Using dual-color fluorescence correlation spectroscopy (FCS) as well as total internal reflection fluorescence (TIRF) microscopy, we show that after fast RNA cleavage the Csm complex rapidly releases internal RNA fragments while it retains both the 3′- and the 5′-ends of the RNA over a timescale of approximately 80 minutes. Using a continuous fluorescent DNA degradation assay, we observed that the RNA-activated ssDNase activity ceases at the long timescale. This implies that after fast activator RNA cleavage, the bound RNA ends provide a molecular memory to keep the Csm complex in the activated state. We further show that upon Csm activation the complex remains capable of RNA turnover, i.e. of ongoing degradation of target RNA. This provides a rather fixed time window for the complex activation independent of the number of available target molecules. Finally, careful modelling of the DNA degradation kinetics allowed developing a quantitative kinetic scheme revealing how the Csm DNase and the RNase activities are coupled.

## Materials and methods

### Construction of expression and substrate plasmids

A TEV protease recognition site was inserted into pCsm2-Tag plasmid (10) between *Streptococcus thermophilus* DGCC8004 *csm2* gene and fused N-terminal StrepII-tag to yield pCsm2-TEV-Tag. For this pCsm2-Tag was PCR amplified, mixed with a splint oligo carrying TEV recognition site insertion and recircularized by Gibson assembly using NEBuilder HiFi DNA Assembly Master Mix (New England Biolabs).

For the production of RNase-dead Csm we substituted wt *csm3* gene in pCas/Csm_ΔCsm6’ΔCsm6 plasmid (50), carrying *S. thermophilus* DGCC8004 *cas6*, *cas10*, *csm2*, *csm3*, *csm4* and *csm5* genes, with D33A *csm3* mutant from pCas/Csm_D33A (10). For this we double-cut pCas/Csm_ΔCsm6’ΔCsm6 with REase Mva1269I and gel-purified the linear fragment. The corresponding insert fragment with *csm3* D33A mutations was PCR amplified from pCas/Csm_D33A. Both DNA fragments were subjected to Gibson assembly to reconstitute pCas/Csm_ΔCsm6’ΔCsm6_Csm3D33A.

To obtain plasmids encoding mismatched RNA substrates we used the pUC18_S3/1 plasmid (10). Using this plasmid as a PCR template, a linear DNA fragment excluding crRNA-complementary S3/1 target region was amplified. Linear DNA was assembled into a plasmid by Gibson assembly using annealed oligos containing 9 nt mismatches to 5′- or 3′-parts of S3 spacer sequence or inverting the whole S3/1 target sequence yielding, respectively, pUC_S3/1_5′mm, pUC_S3/1_3′mm and pUC_S3/1_NS. All mutations and DNA substitutions were confirmed by Sanger sequencing.

### Preparation of Csm complexes

The pCas/Csm_ΔCsm6’ΔCsm6 or pCas/Csm_ΔCsm6’ ΔCsm6_Csm3D33A plasmid were transformed to *Escherichia coli* BL21(DE3) with pCRISPR_S3, containing five repeats interspaced by four identical spacers S3 (10), and pCsm2N-TEV-Tag for wt or RNase-dead Csm complex production. The expression of Csm complex components was induced by 1 mM IPTG for 3 h at 37°C. Cells were harvested by centrifugation and disrupted by sonication in purification buffer (20 mM Tris–HCl (pH 8.5 at 25°C), 500 mM NaCl, 1 mM EDTA, 7 mM 2-mercaptoethanol) supplemented with 2 mM PMSF. The suspension was cleared by centrifugation and complexes from soluble fraction were captured by StrepII-affinity chromatography using StrepTrap HP (GE Healthcare) columns and eluted using purification buffer with 2.5 mM desthiobiotin. Complexes were subjected to size exclusion chromatography and fractionated according to complex size, which is dependent on crRNA maturation level (10). crRNA from different fractions were extracted by phenol/chloroform and analyzed by electrophoresis under denaturing conditions. Fractions in which fully matured 40 nt crRNA species was dominant were merged and incubated with TEV protease overnight. To discard His-tagged TEV protease and uncleaved StrepII-tagged complexes the mixture was subjected to HisTrap (GE Healthcare) and StrepTrap columns. The flow-through was collected, dialyzed against storage buffer (10 mM Tris–HCl (pH 8.5), 300 mM NaCl, 1 mM DTT, 0.1 mM EDTA, 50% (v/v) glycerol), and stored at –20°C.

### RNA substrates

RNA substrates (Supplementary Table S1) were produced by *in vitro* transcription. For this DNA matrices were amplified from pUC18_S3/1, pUC_S3/1_5′mm, pUC_S3/1_3′mm and pUC_S3/1_NS using appropriate DNA primers encoding a flanking T7 RNA polymerase promoter sequence at 5′-end of desired RNA coding region. Purified PCR products were used in the *in vitro* transcription reaction to obtain RNA substrates using TranscriptAid T7 High Yield Transcription Kit (Thermo Scientific) according to manufacturer recommendations. RNAs were purified and concentrated using spin columns. For RNA binding experiments the substrates were 5′-labeled with [γ^32^P]-ATP (PerkinElmer) and T4 PNK (Thermo Scientific) and gel purified.

The internally ATTO647N-labeled RNA substrate was purchased from Eurofins Genomics.

### Electrophoretic mobility shift assay

Substrate binding assays were performed as described earlier (10) with minor modifications. 50 pM of radiolabeled substrate was incubated with 0.1–100 nM of wt Csm complex in a binding buffer (1× TAE, 10% (v/v) glycerol, 0.1 mg/ml BSA) for 10 min at room temperature. Reaction mixtures were analyzed by electrophoresis on native PAGE and depicted using a phosphorimager. Each assay was performed in duplicate.

### DNA oligonucleotides

Single-stranded DNAs (Supplementary Table S2) were purchased HPLC purified and lyophilized from Eurofins Genomics. Pre-hybridized RNA and DNA strands were annealed by slow cooling from 95 to 4°C at 1 K/min in 16 mM Tris-acetate, 33 mM K-acetate and 5 mM EDTA.

### FCS measurements

Time-resolved fluorescence correlation spectroscopy (FCS) measurements were carried out on a Microtime 200 setup (PicoQuant) in time-correlated single photon counting (TCSPC) mode. Samples were excited in a confocal volume by two lasers at 532 nm and 638 nm with a power after all optical filters of 12.9 and 13.4 μW, respectively. The lasers were operated in pulsed interleaved excitation (PIE) mode with pulse widths of 50 ps at a repetition rate of 20 MHz. Reactions were performed in a droplet of 36 μl volume that was restrained by parafilm on cleaned cover slides. Cover slides were sonicated for 10 min in acetone, 10 min in isopropanol and 20 min in 5 M KOH and afterwards thoroughly washed with milli-Q water and blown dry with nitrogen. Reactions were started by adding 3 μl of solution containing the divalent ions to 33 μl of solution containing all other reagents. All FCS measurements were performed in 33 mM Tris-acetate, 66 mM K-acetate, 0.1 mg/ml BSA, 0.25 U/μl Thermo Scientific™ RiboLock RNase Inhibitor and 0.5 mM MnCl_2_ at 25°C.

### Correlation trace fitting

All FCS measurements were analyzed using PAM (PIE analysis with MATLAB), which is a software package that allows quantitative analysis of fluorescence microscopy data obtained using pulsed interleaved excitation (51). Within the software, the correlation function ${G}_{ij}( \tau )$ is calculated according to:


(1) \begin{eqnarray*}{G}_{ij}\left( \tau \right) = \frac{{\left\langle {{F}_i\left( t \right){F}_j\left( {t + \tau } \right)} \right\rangle }}{{\left\langle {{F}_i\left( t \right)} \right\rangle \left\langle {{F}_j\left( t \right)} \right\rangle }},\end{eqnarray*}

where $\langle {{F}_i( t )} \rangle$ and $\langle {{F}_j( t )} \rangle$ are the time averaged fluorescence signals of the two fluorescent channels $i$ and $j$, and ${F}_i( t )$ and ${F}_j( {t + \tau } )$ are the fluorescence intensities at a given time-point ($t$) and the time-point shifted by the lag time ($t + \tau$). By setting $i = j$ (i.e. single-color FCS), one obtains the autocorrelation function, while for $i \ne j$ (i.e. dual-color FCS), one obtains the cross-correlation function. Exported correlation curves were then plotted and analyzed using self-developed Python (version 3.8) scripts and fitted by diffusion models (see Supplementary Note S1) using a nonlinear least-squares method (‘curve_fit’), which is part of the SciPy package (52).

### TIRF experiments

The total internal reflection (TIRF) microscopy experiments were conducted using a custom-built prism-based TIRF setup. The setup employs a Nikon TI2 eclipse microscope equipped with a Plan Apo 60x water immersion objective. The TIRF field was generated above a glass surface within a self-constructed microfluidic flow cell using a 640 nm laser.

The flow cell was assembled using parafilm sandwiched between a cleaned and a functionalized glass coverslide (Menzel). To prepare the clean coverslide, the glass slide was sonicated in acetone and isopropanol, followed by rinsing with Milli-Q water and nitrogen drying. To prepare the functionalized coverslide, the glass slide was passivated through silanization and functionalized with biotin. To this end, the glass slides were first cleaned by sonication in acetone. Following a further sonication step in KOH (5 M), the slides were rinsed with deionized water and MeOH before blowing them dry with nitrogen. For passivation, the slides were first incubated in 150 ml MeOH, 7.5 ml acetic acid and 1.5 ml aminopropylsilane. The glass slides were then coated with a mixture of mPEG (Rapp Polymere) and biotinylated mPEG (50:1) dissolved in 100 mM sodium bicarbonate at pH 8.5 and incubated overnight. The slides were stored under vacuum conditions at − 20°C.

To immobilize the molecules on the surface, we sequentially introduced into the flow cell 100 nM streptavidin, 100 nM biotinylated single-stranded DNA, 2 nM RNA hybridized to an equal amount of AlexaFluor647-double-labeled DNA, and 10 nM Csm. To start the reaction, we introduced the imaging buffer (2 mM Trolox, 5 mM PCA, 75 nM PCD, 33 mM Tris-acetate, 66 mM K-acetate, 0.1 mg/ml BSA, 0.25 U/μl Thermo Scientific™ RiboLock) together with 0.5 mM MnCl_2_, at 25°C. Afterward, we recorded images in 20-second intervals with an exposure time of 100 ms.

Image analysis was performed using Fiji 2.14.0 imageJ 1.54f, which involved initial image smoothing, drift correction using the *Fast4DReg* plugin (53), and spot detection and tracking with the *spot intensity analysis* plugin. The obtained trajectories were analyzed using custom python (version 3.8) scripts. We first applied a 3-point moving average, followed by normalizing the trajectory against the first 10 data points. Afterward, we recorded the time when the signal dropped below 10% of the initial intensity to generate the survival probability.

### Bulk fluorescence measurements

All bulk fluorescence measurements were performed in a temperature-controlled Cary Eclipse fluorescence spectrometer (Agilent) in 150 μl cuvettes (Hellma Analytics), in buffer containing 33 mM Tris-acetate, 66 mM K-acetate, 0.1 mg/ml BSA, 0.25 U/μl Thermo Scientific™ RiboLock RNase Inhibitor and 0.5 mM MnCl_2_ at 25°C. The excitation wavelength was set to 495 nm and the emission wavelength was recorded at 518 nm, both with a slit width of 10 nm. The photomultiplier tube voltage was set to 550 V. 100 μl of RNA and DNA solution were always added first and pre-incubated for 300 s. Afterwards 100 μl of solution containing the Csm complex was quickly pipetted to start the reaction. Datapoints were recorded approximately 10 s after reaction start in 1-second intervals. The fluorescence signal was transformed to cleaved DNA using a linear scaling factor, which was obtained by measuring the saturated signal of known amounts of DNA being fully cleaved by micrococcal nuclease (New England Biolabs).

### Time trace fitting

Fluorescence time traces were analyzed using self-developed Python (version 3.8) scripts. All fits were performed using a nonlinear least-squares method (‘curve_fit’), which is part of the SciPy package (52).

To fit the rate equation model, a set of ordinary differential equations was solved using the ‘odeint’ function (which is also part of the SciPy package) and the solution was then used in the ‘curve_fit’ routine.

## Results

### Dual-color FCS reveals long-lasting retention of the RNA ends

Previously it has been shown (7), that despite rapid target-specific RNA cleavage by the Csm complex, the downstream single-stranded DNase activity persists over a considerably longer duration. Notably, target RNA cleavage yields short (6 nt long) internal fragments and longer terminal fragments located at both ends of the complex (10) (see Figure 1B). To comprehend the spatiotemporal control of the ssDNA cleavage activity by Csm, we systematically investigated the release of the different RNA fragments from the Csm complex. Therefore, we utilized a dual-color FCS setup in pulsed interleaved excitation (PIE) mode (54), which allows monitoring the diffusion properties of multiple biomolecules simultaneously. Within the setup, freely diffusing and fluorescently labeled RNA molecules were alternatingly illuminated with green and red laser pulses within the diffraction limited spot of the laser focus and the resulting fluorescence emission was measured in separate channels. Using low substrate concentrations (pico to nanomolar) ensured that only a small number of fluorophores were excited simultaneously in the confocal volume (a few femtoliters). By recording the fluorescence signal at high temporal resolution and processing the resulting fluctuation trajectories using correlation analysis, the proportion of released RNA fragments can be monitored in real-time (55,56).

**Figure 2.**
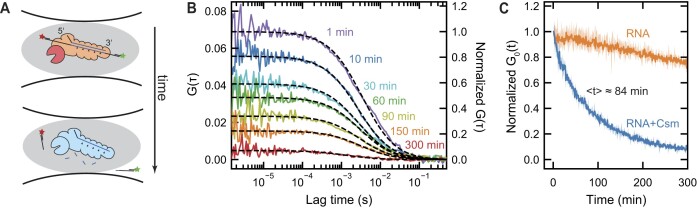
Fluorescence cross-correlation spectroscopy reveals retention time of the RNA ends. (**A**) Scheme of the Csm complex diffusing through the confocal excitation volume. For intact target RNA, the ends diffuse jointly through the focus yielding a significant cross-correlation between green (ATTO-532) and red (ATTO-647N) fluorophore emission. Reaction initiation leads to RNA cleavage and consecutive release of the target RNA, resulting in a spatial separation of the dyes and a drop in cross-correlation. (**B**) Representative fluorescence cross-correlation curves as function of lag time measured for the activating target RNA S3/a at different time points of the reaction (colored solid lines). Each correlation curve was calculated from a one-minute time interval of the fluorescence signal. Fits of the data to a simple 3D diffusion model are shown as dashed lines. Absolute cross-correlation values are given by the left axis and normalized values by the right axis. (**C**) Normalized time-dependent cross-correlation amplitudes for activating RNA target S3/a in absence (orange) and presence (blue) of Csm complex. The kinetics were obtained from the amplitude of cross-correlation curves calculated from consecutive 1-min intervals of the fluorescence signal. Solid lines indicate the mean of the signal for three repeated measurements, while colored areas indicate the range between measured minimum and maximum value. All curves were normalized to 1 at zero time before averaging. The approximate timescale of 84 minutes for RNA end release was obtained from an exponential fit to the fraction of retained RNA ends (see Supplementary Figure S3). Reactions were initiated by adding 0.5 mM MnCl_2_ to the solution containing 2 nM activating target RNA S3/a and 10 nM Csm.

First, we aimed to determine the duration at which the cleaved RNA end fragments remain bound to the Csm complex. To achieve this, we designed 180 nt long RNA substrates (Supplementary Figure S1, Supplementary Table S1) to be used for fluorescent labeling and assessed their binding to Csm (Supplementary Figure S2). For labeling, we annealed 5′-ATTO647N-labeled and 3′-ATTO532-labeled DNA oligonucleotides, each 30 nt in length, to the 3′- and 5′-ends of the RNA substrates, respectively (see Supplementary Figure S1, Supplementary Table S2). By probing the probability that the two labels diffuse together through the confocal volume, the integrity of the RNA substrate was monitored. Before each experiment, Csm complexes were pre-bound to the RNA substrates in the absence of divalent ions, such that no RNA cleavage occurred. Then, the reaction was initiated by the addition of divalent ions. We first measured the RNA cleavage product release kinetics for the activating target RNA S3/a substrate. As long as the complexes were bound to the RNA, they diffused together with both labels through the confocal volume, such that the fluorescence fluctuations in both channels were similar (i.e. correlated). Upon RNA cleavage and release by the Csm complex, the fluorophores became separated such that they travelled independently through the laser focus and the fluorescent signals became uncorrelated (Figure 2A). To quantify the extent of joint vs. independent diffusion of the RNA ends through the laser focus, we calculated cross-correlation functions (see Methods) between the two fluorescence signals as function of the lag time (solid lines in Figure 2B). The measured cross-correlation functions were well fitted by a 3-dimensional diffusion model (dashed lines in Figure 2B, see Supplementary Note S1). This yielded the mean diffusion time ${\tau }_D$ of the intact RNA through the confocal volume as well as the amplitude of the cross correlation at zero lag time. Notably, ${\tau }_D$ stayed rather constant throughout the time course of the reaction, indicating that the diffusive properties of the observed species did not change. The amplitude of the cross-correlation curve is proportional to the concentration of joint diffusion of the RNA ends through the focus, i.e. the amount of Csm with both RNA ends bound. Plotting the cross-correlation amplitude as function of the reaction time, revealed an exponential decay of the signal on the time-scale of an hour (blue curve in Figure 2C). Notably, the correlation amplitude approached zero over time, indicating full RNA cleavage and release of almost all RNA ends. As a control, we measured the same RNA substrate in the absence of Csm. The obtained trajectory showed only a small decrease during the observed time-scale (orange in Figure 2C), which we attributed to evaporation effects, spontaneous RNA degradation and photobleaching.

To extract quantitative parameters, we normalized the cross-correlation amplitude in the presence of Csm by the amplitude measured in absence of Csm, which could be described by a single exponential decay (see Supplementary Figure S3):


(2) \begin{eqnarray*}R\left( t \right) = \left( {1 - {R}_0} \right){e}^{ - t/\left\langle t \right\rangle } + {R}_0,\end{eqnarray*}

with ${R}_0$ being the remaining amplitude at infinite time, reflecting uncleaved/unreleased RNA, and $\langle t \rangle$ being the characteristic time-scale of the RNA end release. This yielded a mean release time of $\langle t \rangle = ( {84 \pm 1} )$ min for the release of the RNA ends and a residual amplitude of ${R}_0 = ( {8.7 \pm 0.2} )$ %. Notably, the single exponential character indicates that the observed reaction is of first order and does not involve second order binding steps. Overall, we conclude from the agreement between the experimental data and the intuitive modeling, that the established assay correctly probes the reaction kinetics of the RNA end release.

To gain a deeper understanding of the RNA end release process and to further validate the established method, we conducted a series of control experiments with modified conditions (colored traces in Supplementary Figure S4) in comparison to the standard reaction using the activating RNA target S3/a, Csm, and MnCl_2_ (black traces in Supplementary Figure S4).

First, we omitted the addition of MnCl_2_, for which the cross-correlation amplitude stayed rather constant throughout the time course of the reaction (purple trace in Supplementary Figure S4) demonstrating that MnCl_2_ addition indeed triggers the RNA degradation. Furthermore, we performed measurements employing a Csm RNase-dead mutant (D33A) (10), as an additional control for the requirement of RNA cleavage for the separation of the dye pair. As expected, the cross-correlation amplitude (orange trace in Supplementary Figure S4) stayed rather constant throughout the time course of the reaction.

Next, we investigated the sequence specificity of the RNA cleavage by the Csm complex. To this end, we tested a non-specific RNA substrate NS with only few and separated matches with the crRNA. The cross-correlation amplitude for this non-specific RNA substrate only decreased slightly over the time-course of the reaction (green trace in Supplementary Figure S4), indicating that RNA cleavage requires base pairing of the target RNA with the crRNA, in agreement with a previous studies (10). Additionally, we tested a non-activating target RNA substrate S3/n, which forms eight additional base pairs with the 5′-end of the crRNA and is cleaved by Csm, but does not lead to activation of single-stranded DNA cleavage. Interestingly, the cross-correlation amplitude decreased on the same timescale as the activating target RNA S3/a (cyan trace in Supplementary Figure S4), which suggests a loss of the cross-correlation due to the release of the fragment at the 3′-end and/or a hybridization independent release mechanism of the 5′-end.

Finally, we examined the impact of the cofactors required downstream after Csm activation by RNA binding. First, we performed measurements in the presence of ATP, to investigate whether activation of the cA_*n*_ synthesis by the Cas10 subunit affects the mechanics of the RNA end release. The obtained trajectory (red trace in Supplementary Figure S4) decayed on the same timescale as in the absence of ATP. Additionally, we investigated whether activated single-stranded DNA cleavage by the Cas10 subunit affects the RNA end release. The obtained trajectory (yellow trace in Supplementary Figure S4) decayed again on a similar timescale as in the absence of ssDNA. These observations suggest that the RNA end release mechanism by the Csm complex operates independently of its downstream activities.

Additionally, we utilized Total Internal Reflection Fluorescence (TIRF) microscopy as an alternative to probe the release of the RNA ends from the Csm complex. To this end, we immobilized the activating target RNA substrate S3/a on its 5′ end onto a glass surface using a biotinylated DNA oligonucleotide that binds to streptavidin on the surface (see Supplementary Figure S5A). Furthermore, the 3′-end of the substrate was labeled by hybridizing an Alexa647-labeled DNA oligonucleotide, enabling its detection by imaging in a TIRF microscope. Similar to the dual-color FCS experiments, we pre-incubated the Csm complex with the RNA substrate, initiated the reaction by the addition of divalent ions and recorded fluorescence images every 20 s over the duration of 60 min. Over time, part of the fluorescent spots, each representing a RNA 3′-end, disappeared (Supplementary Figure S5A). We then utilized a spot tracking algorithm to determine the time courses of the intensities of each spot. While some spots retained their intensities over the full duration, some went dark in a sudden intensity drop, representing the release of the RNA ends (see Supplementary Figure S5B) or photobleaching. To separate end release and photobleaching, we repeated the same measurement in absence of Csm, where an end release does not occur. While in the absence of Csm, only 25% of the fluorescence spots disappeared over 60 min (blue line in Supplementary Figure S5C), in the presence of Csm 65% of the spots vanished (orange line in Supplementary Figure S5C) indicating significant end release. To obtain a kinetics for the end release only, we corrected the measured kinetics in presence of Csm using the kinetics measured in its absence by simply dividing the corresponding survival probabilities. The kinetics of RNA end release measured with TIRF microscopy was in good agreement with the kinetics from the dual-color FCS measurements (Supplementary Figure S5D), providing independent and robust support for our findings.

Altogether, we established an assay that correctly probes the RNA end retention by the Csm complex. Our results demonstrate that despite RNA cleavage occurring on the timescale of seconds, the RNA ends are retained on a timescale of an hour. Furthermore, we showed that RNA cleavage is sequence specific and that possible downstream activities do not alter the retention timescale.

### Single-color FCS measurements reveal fast internal fragment release

**Figure 3.**
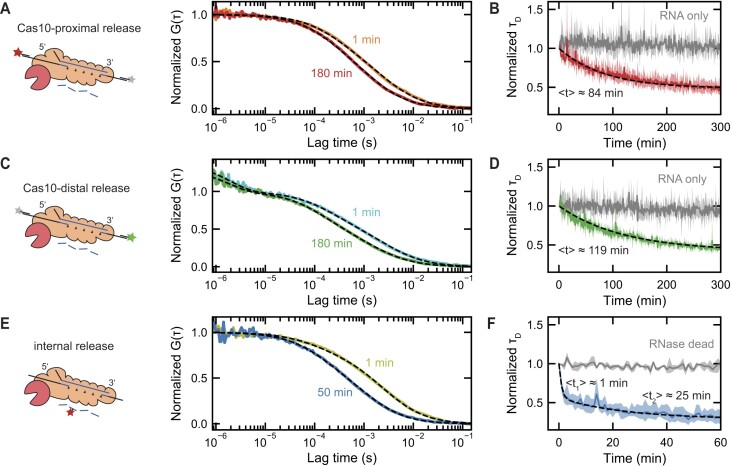
Fluorescence autocorrelation spectroscopy reveals retention time of different RNA fragments. (**A**) Representative autocorrelation curves of the Cas10-proximal end of the activating target RNA S3/a (ATTO-647N label, see sketch in the left) measured at 1 min and 180 min after reaction start (solid lines). (**B**) Normalized diffusion time as function of reaction time for the Cas10-proximal end of the activating target RNA S3/a in the presence (red solid line) and absence (gray solid line) of Csm. An exponential fit to the data (dashed line) provided a mean time for the Cas10-proximal RNA end release of $\langle t \rangle = ( {84 \pm 2} )$ min. (**C**) Representative autocorrelation curves of the Cas10-distal end of the activating target RNA S3/a (ATTO-532 label, see sketch in the left) measured at 1 min and 180 min after reaction start (solid lines). (**D**) Normalized diffusion time as function of reaction time for the Cas10-distal end of the activating target RNA S3/a in the presence (green solid line) and absence (gray solid line) of Csm. An exponential fit to the data (dashed line) provided a mean time for the Cas10-distal RNA end release of $\langle t \rangle = ( {119 \pm 3} )$ min. (**E**) Representative autocorrelation curves of the internally labeled activating target RNA S3/iL (ATTO647N, see sketch in the left), measured at 1 min and 50 min after reaction start (solid lines). (**F**) Normalized diffusion time as function of reaction time for internally labeled activating target RNA S3/iL in the presence of Csm (blue solid line) and the RNase-dead mutant (gray solid line). An exponential fit, employing two decaying species, to the data (dashed line) provided the mean times of the internal fragment release of $\langle {{t}_1} \rangle = ( {1.0 \pm 0.1} )$ min and $\langle {{t}_2} \rangle = ( {25 \pm 4} )$ min. Dashed lines in (A, C, E) show fits to a model including a slowly and a rapidly diffusing species. The diffusion times as function of time were obtained from autocorrelation curves calculated from consecutive 1-min intervals from the signal of one fluorescence channel.

We next aimed to further explore the RNA fragment release and to investigate whether the loss in the cross-correlation amplitude can be attributed to the release of just a single RNA end at one side. To accomplish this, we calculated separate autocorrelation functions (see methods) for either the green (Figure 3A) or the red (Figure 3C) detection channel. The autocorrelation amplitude stayed rather constant in this case. However, a shift of the mean diffusion time of the individual RNA ends through the confocal volume towards lower times was observed. This is consistent with the progressive release of RNA ends from the Csm complexes, since the free RNA ends diffuse faster. For a quantitative description of the autocorrelation curves, we expanded the previous model by including two diffusing species as well as a triplet decay for the green fluorophore (see Supplementary Note S1). We accounted the first species to be the corresponding RNA end bound to Csm, and the second species to be the free RNA end. The model showed excellent agreement with the experimentally determined autocorrelation curves over the full range of lag times (see dashed lines in Figures 3A, C). From the best-fit parameters, we calculated a mean diffusion time of both species as function of the reaction time as a measure for the release of the respective RNA end (Figures 3B, D). The mean diffusion time again showed an exponential decay with time in the presence of Csm, while in its absence only an insignificant change could be observed. We fitted a single exponential decay (Equation (1)) to the obtained time courses and obtained a mean release time of $( {84 \pm 2} )$ min and $( {119 \pm 3} )$ min for the Cas10-proximal and Cas10-distal RNA end, respectively. Remarkably, the timescales observed for the release of the individual RNA ends roughly agree with the timescale obtained by analyzing the cross-correlation signal, which represents the combined release of both ends. Notably, if we were to assume independent releases of both RNA ends, the expected mean time for the loss in cross-correlation would be around 50 min. The observed longer time thus suggests that the release of both RNA ends occurs with a certain degree of cooperativity. This corresponds to a coordinated mechanism for the RNA fragment release, where the liberation of one end is facilitated by the release of the other end.

Next, we were interested whether the short (6 nt) internal fragments that are produced during RNA cleavage are released on the same long timescale. To probe this, we employed a 68 nt long target strand (see Supplementary Figure S6), that carried an internal ATTO647N fluorophore. The label is located such that it lies within the binding region of Csm3.3 in the ternary Csm complex. Upon target RNA cleavage, short ATTO647N-fragments are produced. After release (see Figure 3E), these fragments will diffuse significantly faster than when bound to the complex. Similar to before, we measured the fluorescence signal after the addition of divalent ions. Calculating autocorrelation functions revealed that the ternary complex with the internally labeled RNA, initially exhibits a comparable diffusion behavior as the ternary complex with the end labeled target RNA (see yellow curve in Figure 3E). During the reaction, the characteristic diffusion time of the internal label decreased significantly faster than that of the end labels (see blue curve in Figure 3E). To quantify our observations, we again fitted a model function incorporating diffusion in three dimensions through a Gaussian-shaped detection volume as well as two RNA species (see Supplementary Note S1). The resulting fits reproduced the obtained autocorrelation curves well (see dashed lines in Figure 3E). Plotting the resulting mean diffusion times versus time showed a bi-phasic decay (see blue trace in Figure 3F) being considerably faster than the RNA end release. As a control, we performed the same experiment employing the RNase-dead mutant. As expected, the autocorrelation amplitude stayed constant throughout the time course of this reaction (see gray trace in Figure 3F) since short internal RNA fragments were not produced. We fitted a bi-phasic decay of the diffusion time, by using a sum of two exponential decays:


(3) \begin{eqnarray*}\tau \left( t \right) = {A}_1{e}^{ - t/{{\left\langle t \right\rangle }}_1} + {A}_2{e}^{ - t/{{\left\langle t \right\rangle }}_2} + {\tau }_0,\end{eqnarray*}

with ${\tau }_0$ being the diffusion time of the short fragments and ${A}_1 = 1 - {A}_2 - {\tau }_0$ and ${A}_2$ being the amplitudes of the species exhibiting the mean decay times ${\langle t \rangle }_1$ and ${\langle t \rangle }_2$, respectively. The resulting fit described the data (see dashed line in Figure 3F) and provided the mean decay times ${\langle t \rangle }_1 = ( {1.0 \pm 0.1} )$ min and $\langle {{t}} \rangle_2 = ( {25 \pm 4} )$ min. We attribute the fast phase to full target RNA cleavage and the slow phase to intermediate cleavage products, which remain slightly longer.

Overall, evaluation of the diffusion times of the labeled RNA fragments confirmed that the RNA ends are retained for an elongated period of time (hours) while it revealed that the short internal fragments are released on a much faster timescale (minutes).

### Single-stranded DNA cleavage activity correlates with retention of the RNA ends

**Figure 4.**
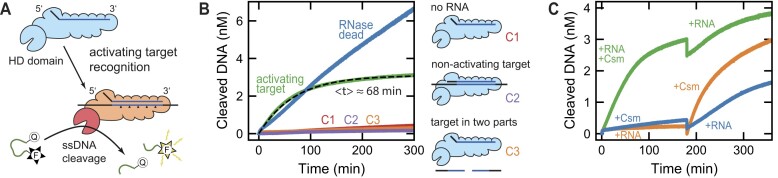
Bulk fluorescence measurements reveal the duration of the RNA-activated DNA cleavage. (**A**) Scheme of the Csm complex binding to an activating target RNA, which initiates the ssDNA cleavage by Cas10. ssDNA cleavage is monitored by a fluorophore-quencher pair bound to the ends of a 6 nt oligonucleotide. ssDNA cleavage separates the labels and leads to a fluorescence increase. (**B**) Cleaved ssDNA as function of time measured for activating target RNA S3/a added to either the Csm complex (green line) or its RNase-dead mutant (blue line). Additionally, control measurements in the absence of RNA (red line, C1), in the presence of the non-activating target RNA S3/n (purple line, C2) and of the activating target split into two RNA fragments (orange line, C3) are shown. An exponential fit to the time trace of the Csm complex with the activating target (black dashed line) yielded a decay time of $\langle t \rangle = ( {68 \pm 1} )$ min. Shown traces represent the mean of three repeated measurements. Reactions were initiated by adding 2 nM Csm (or RNase-dead mutant) to 2 nM target RNA, 0.5 mM MnCl_2_ and 100 nM ssDNA (or 200 nM ssDNA in case of the RNase-dead mutant). (**C**) Cleaved ssDNA as function of time measured after adding Csm complex and activating target RNA S3/a at different time points to the solution containing 0.5 mM MnCl_2_ and 100 nM ssDNA. For each addition, either 2 nM Csm and/or 4 nM RNA were added. Shown are an addition of Csm and RNA at $t = 0$ min followed by an extra addition of RNA at $t = 180$ min (green trace), an addition of Csm at $t = 0$ min followed by RNA addition at $t = 180$ min (blue trace) and an addition of RNA at $t = 0$ min followed by Csm addition at $t = 180$ min (blue trace). Shown traces represent the mean of three repeated measurements.

Having resolved the timescales of the RNA fragment release, we next aimed to investigate the RNA-dependent temporal control of the ssDNA cleavage by the HD domain of the Cas10 subunit. To this end, we employed a short (6 nt long) single-stranded DNA oligonucleotide (see Supplementary Table S2), labeled at the 5′-end with a fluorophore (6-FAM) and at the 3′-end with a fluorescence quencher (BHQ-1). Due to the proximity of the labels, the fluorescence emission is quenched. However, upon DNA cleavage induced by the Csm complex, the quencher becomes spatially separated from the fluorophore, resulting in an observable increase in fluorescence emission (Figure 4A). To track the ssDNA cleavage activity of the Csm complex, we monitored the time-dependent fluorescence signal after the addition of unlabeled RNA target strands, utilizing a fluorescence spectrometer.

We first measured the DNA cleavage activity for the activating target RNA S3/a (green trace in Figure 4B). The resulting time trajectory exhibited a fluorescence increase, indicating ssDNA cleavage activity, which decayed over the course of an hour. To relate the measured fluorescence signal to the amount of cleaved DNA, we incubated known amounts of ssDNA with a highly efficient nuclease and mapped the fluorescence signal after completion of the reaction (see Supplementary Figure S7). This provided a linear scaling factor between the two quantities. The quantification revealed that there was only a moderate ssDNA turnover per Csm complex at the applied concentrations. The acquired ssDNA cleavage kinetics $F( t )$ was well described by a single exponential fit:


(4) \begin{eqnarray*}F\left( t \right) = A\left( {1 - {e}^{ - t/\left\langle t \right\rangle }} \right),\end{eqnarray*}

with $A$ being the total cleaved ssDNA and $\langle t \rangle$ being the mean time of the reaction. It provided a mean reaction time of $\langle t \rangle = ( {68 \pm 1} )$ min, which agrees with the timescale observed for the RNA end release.

To further validate our ssDNA cleavage assay, we performed a number of different control experiments. Measurements in the absence of target RNA (red trace in Figure 4B), the presence of the non-activating target RNA S3/n (purple trace in Figure 4B) as well as the activating target RNA split into two fragments (orange trace in Figure 4B) revealed only a minor ssDNA cleavage activity in the background. Thus, recognition of the activating target RNA as a full piece is required to activate ssDNA cleavage by the Cas10 subunit. Furthermore, we performed ssDNA cleavage measurements using RNase-dead Csm and activating target RNA S3/a (blue trace in Figure 4B). Pronounced ssDNA cleavage activity was observed that in contrast to wt Csm continued almost unchanged during the full measurement period. Considering that the activity of the Csm complex decayed on the same timescale as the release of RNA ends (84 min), this suggests that RNA cleavage and the subsequent end release establish the temporal control of the DNase activity.

In order to probe whether the RNA release is also employed to discriminate mutated RNA targets, we conducted experiments using two target RNA substrates containing 9 base pair mismatches with the crRNA at either the 5′-end or the 3′-end of the crRNA (see Supplementary Figure S1 and Supplementary Table S1). The fluorescence measurements showed that neither of these mismatched substrates led to the activation of the DNase activity of the wt Csm complex (Supplementary Figure S8B) or the Csm RNase-dead mutant (Supplementary Figure S8C), in agreement with previous reports (7,37). In addition, we performed dual-color FCS experiments to measure the release of the RNA ends during the reaction (Supplementary Figure S8D). Surprisingly, for mismatches at the 3′-end of the crRNA the release was slightly slower ($\langle t \rangle = ( {112 \pm 1} )$ min), while for mismatches at the 5′-end the release was much faster ($\langle t \rangle = ( {34 \pm 1} )$ min), as compared to the activating target ($\langle t \rangle = ( {84 \pm 1} )$ min). However, the release for both mismatched substrates was still occurring on a rather long timescale of 10s of min. As the target RNA is still much more rapidly cleaved for both types of mismatches (10), the Cas10 subunit activation can thus not be solely attributed to the retention of the RNA ends, but has to involve further steps following full substrate binding (see Discussion).

After establishing a coupling between single-stranded DNase activity and RNA end binding, we were interested whether the DNase activity could be reinitiated through the reintroduction of intact target RNA. Therefore, we first determined that the active enzyme concentration matches the employed RNA concentration, by evaluating the steady state kinetics for increasing enzyme concentrations (see Supplementary Figure S9). Afterwards, we performed experiments of adding RNA and Csm complex at different time points (see Figure 4C). First, we initiated ssDNA cleavage activity by adding RNA and Csm at the same time, which reproduced the previous observations (green trace in Figure 4C). After the ssDNA cleavage slowed down significantly ($t = 180$ min), we added again the same amount of RNA. The ssDNA cleavage became again faster and saturated after a similar timescale as before. This observation demonstrates a reactivation of the DNase activity of the Csm complex upon the introduction of intact target RNA. Notably, the reactivation of the ssDNA activity was markedly reduced compared to the initial activation. To explore whether this decline in DNase activity results from RNA-unrelated complex deactivation in solution, we conducted measurements where we introduced the Csm complex first, followed by RNA addition after 180 min (blue trace in Figure 4C). The resulting ssDNA activity appeared to be reduced as for the reactivation. As a further control, we added the RNA first, followed by the addition of the Csm complex after a 180 min interval (orange trace in Figure 4C). The resulting ssDNA activity was similar to the activity for simultaneous addition of Csm complex and RNA. This indicates that the RNA remains structurally intact and does not suffer from non-specific degradation throughout the observed timescale. Consequently, we attributed the reduced ssDNA cleavage activity during the second activation to a non-specific Csm deactivation, such as complex aggregation or denaturation. This may also explain the slightly lower duration for the DNase activity compared to the timescale of the RNA end release as shown before (68 min versus 84 min).

In summary, we have disclosed a correlation between the timescale of the single-stranded DNase activity (as observed through bulk fluorescence measurements) and the timescale of RNA end release (quantified via fluorescence correlation spectroscopy). Furthermore, we have demonstrated the important role of RNA cleavage and release in governing the temporal control of the DNase activity. Additionally, our findings show that the Csm complex is capable to undergo multiple turnovers of ssDNA degradation, which can be reactivated upon the addition of intact RNA.

### Csm executes rapid turnover on RNA without altering the duration of the DNase activity

**Figure 5.**
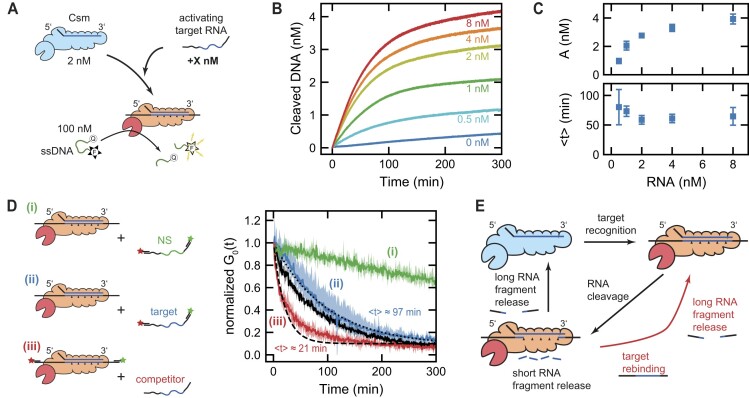
Fast RNA turnover does not influence the duration of the DNase activity. (**A**) Scheme illustrating the bulk fluorescence measurements of the DNase activity for increasing RNA concentration while the initial concentrations of Csm and double-labeled ssDNA were kept constant at 2 and 100 nM. (**B**) Bulk fluorescence measurements of the DNase activity for activating target RNA S3/a concentrations varying between 0 and 8 nM. Shown traces are the mean of three repeated measurement. (**C**) Total cleaved ssDNA during activation and mean time $\langle t \rangle$ of the reactions obtained from fitting the traces in B (see main text). (**D**) Dual-color FCS measurements for the configurations (i), (ii) and (iii). (**i**) Preincubation of 2 nM Csm complex with 2 nM unlabeled activating target RNA followed by the addition of fluorescently labeled non-specific RNA. (**ii**) Preincubation of 2 nM Csm complex with 2 nM unlabeled activating target RNA followed by the addition of fluorescently labeled activating target RNA. An exponential fit to the experimental data (black dotted line) provided a mean reaction of $\langle t \rangle = ( {97 \pm 1} )$ min. (**iii**) Preincubation of 2 nM Csm complex with 2 nM labeled activating target RNA followed by the addition of an excess (50 nM) of unlabeled activating target RNA as competitor. An exponential fit to the experimental data (black dashed line) provided a mean reaction of $\langle t \rangle = ( {21 \pm 1} )$ min. Additionally shown for reference are measurements employing 2 nM activating target RNA and 10 nM Csm (black trace; $\langle t \rangle = ( {84 \pm 1} )$ min; see blue trace in Figure 2C). Solid lines indicate the mean of three repeated measurements, while colored areas represent minimum and maximum values. All reactions were started by adding 0.5 mM MnCl_2_. (**E**) Scheme for the RNA turnover by the Csm complex. Following RNA target recognition, the DNase activity is enabled. Release of short internal RNA fragments allow binding of new target RNA that subsequently replaces the initially bound RNA ends such that a RNA turnover is achieved. Upon full RNA degradation, the final RNA ends are released and the DNase activity is terminated. Triangles denote Csm RNA cleavage positions.

Having shown that the ssDNase activity of the Csm complex can be reactivated by additional target RNA, thus enabling RNA turnover, we were next interested whether the ssDNase activity can be extended by providing an excess of RNA. To this end, we performed bulk fluorescence measurements as previously, but kept the Csm and ssDNA concentrations constant, while varying the activating target RNA S3/a (see Figure 5A). For substoichiometric amounts of RNA compared to Csm, the total degraded ssDNA increased as we increased the RNA concentration in agreement with an increasing saturation of the available complexes. Interestingly, when the amount of RNA exceeded the Csm complexes the total amount of cleaved ssDNA increased only slightly. To quantify these observations, we fitted the ssDNA cleavage kinetics with an exponential and an additional background with constant rate:


(5) \begin{eqnarray*}F\left( t \right) = A\left( {1 - {e}^{ - t/\left\langle t \right\rangle }} \right) + ct,\end{eqnarray*}

with $A$ being the cleaved ssDNA during activation, $\langle t \rangle$ being the mean time of the reaction and *c* being the background rate. The fit described the cleavage kinetics for all tested RNA concentrations (see Supplementary Figure S10). Importantly, it provided the total cleaved ssDNA and the mean activation time (Figure 5C). Interestingly it revealed that the duration of the DNase activity remained rather constant over all tested RNA concentrations and that the total cleaved ssDNA started to become saturated when exceeding the equimolar RNA:Csm ratio. These observations suggest that the complex is capable of performing a fast turnover on RNA, while bound RNA ends still determine the duration of the DNase activity.

As an additional test of this idea, we investigated whether activated Csm complexes can be deactivated by non-activating target RNA after it has been activated. To this end, we pre-incubated Csm complexes with activating target RNA S3/a, added upon the reaction initiation with MnCl_2_ an excess of non-activating target RNA S3/n and probed the ssDNase activity (Supplementary Figure S11). The obtained fluorescence trajectories exhibited a significantly reduced mean time (32 ± 1 min) as well as amplitude of the ssDNase activity compared to the absence of non-activating target RNA. This suggests that the exchange of the RNA during the turnover even includes the RNA ends, thus leading to a deactivation of the complex.

To probe the suggested rapid turnover directly on the RNA level, we performed dual-color FCS measurements. We pre-incubated Csm with activating target RNA S3/a, added additional RNA substrates upon reaction initiation with MnCl_2_ and probed the separation of the ends of a labeled RNA substrate. To reveal a sequence-specific RNA turnover, we preloaded the complex with unlabeled activating target RNA S3/a, and added a double-labeled RNA with either a non-specific ((i) in Figure 5D) or an activating target sequence ((ii) in Figure 5D). For the non-specific RNA, the cross-correlation amplitude decreased only slightly during the duration of the reaction (green trace in Figure 5D). In contrast, the additionally added double labeled activating target RNA was cleaved and released on the same timescale ($\langle t \rangle = ( {97 \pm 1} )$ min) as the pre-bound RNA before (compare blue and black traces in Figure 5D), indicating a specific and quick turnover of the RNA, with subsequent retention of the RNA ends. To show that the pre-bound RNA was indeed quickly replaced, we additionally performed measurements in which Csm was pre-incubated with double-labeled activating target RNA S3/a and an excess of unlabeled activating target RNA S3/a was added upon reaction initiation as a strong competitor ((iii) in Figure 5D). In this case, the cross-correlation amplitude of the pre-bound RNA decayed significantly faster ($\langle t \rangle = ( {21 \pm 1} )$ min) compared to the absence of competing activating target (compare red and black trace in Figure 5D) confirming again a fast RNA turnover. Of note, this time scale consistently agrees with the mean time of the ssDNase activity after adding non-activating target RNA (Supplementary Figure S11).

Using bulk fluorescence and dual-color FCS measurements, we could thus show that the Csm complex is capable of performing a rapid turnover of specific target RNA, while maintaining its DNase activity. Mechanistically, this can be explained by a simple reaction scheme for the RNase activity of the Csm complex (see Figure 5E). Herein, target RNA is quickly cleaved and the short fragments are rapidly released, while the long RNA end fragments are retained. The region of unpaired crRNA can base-pair with a new target RNA resulting in the release of the bound RNA ends, without interfering with the DNase activity. Finally, after all available RNA strands have been cleaved, the complex maintains its DNase activity until the last RNA ends are released.

### A simplified kinetic model for the RNA-dependent ssDNA cleavage by the Csm complex

Lastly, we aimed to characterize the kinetics of the DNase activity of the Csm complex to understand the ssDNA binding and the cleavage dynamics by the Cas10 subunit. For this purpose, we conducted bulk fluorescence measurements, akin to our previous approach, by varying the concentration of the short double-labeled ssDNA oligonucleotide.

**Figure 6.**
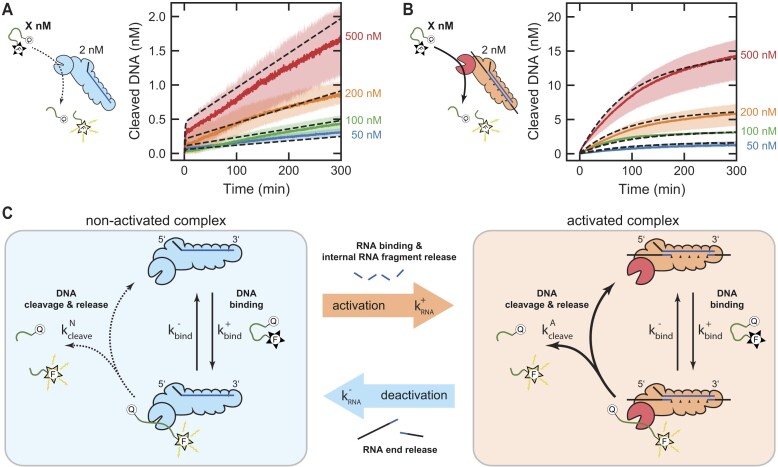
A simple kinetic model for the RNA-dependent DNase activity of the Csm complex. (**A, B**) Bulk fluorescence measurements displaying the single-stranded DNase activity of the Csm complex across a range of ssDNA concentrations. The measurements were conducted using 2 nM Csm in the presence of 2 nM activating target RNA (B) and in its absence (A). Solid lines indicate the mean of three repeated measurements, while colored areas represent minimum and maximum values. Additionally shown is a global fit (dashed lines) of a simplified reaction scheme (see Supplementary Note S2). (**C**) Reaction scheme depicting the RNA-dependent DNase activity of the Csm complex. Both, the non-activated complex (not bound to any RNA fragments) and the activated complex (bound to RNA fragments), share the same single-stranded DNA binding kinetics, while differing in DNA cleavage activity. The single-stranded DNA initially binds to both complex entities in a reversible step and is subsequently cleaved and released in an irreversible step. Releasing the cleaved DNA enables the complex to bind new DNA molecules. The complex is activated by binding and cleavage of target RNA molecules. During activation, the short internal RNA fragments are rapidly released. Upon the release of the long RNA end fragments, the complex is deactivated.

Initially, we conducted measurements with Csm but in the absence of RNA, to characterize the non-specific DNase background activity. The obtained fluorescence traces (see Figure 6A) exhibited a rapid initial phase followed by an approximately linear increase in time. Remarkably, the amplitude of the fast initial phase and the slope of the linear rise increased with increasing ssDNA concentration. Subsequently, we performed measurements with Csm and an equimolar amount of activating target RNA S3/a, to characterize the RNA-dependent temporal regulation of the DNase activity. The obtained fluorescence trajectories (see Figure 6B) demonstrated a significantly elevated DNase activity compared to the measurements in the absence of RNA. As before, the activity increased with increasing DNA concentration. Remarkably, the ssDNase activity decayed on a similar timescale independent of the DNA concentration in agreement with the idea that it is regulated by the RNA end release. Furthermore, the amount of cleaved DNA indicated multiple turnovers of ssDNA on the complex. Notably, the traces also showed a fast initial increase, with a similar amplitude to the measurements in the absence of RNA. We attribute this initial fluorescence increase to the binding of the complex to DNA, resulting in the separation of the dyes without the occurrence of cleavage.

For a quantitative understanding of the data, we devised a simplified kinetic model that captures the main aspects of the RNA-dependent DNase activity of the Csm complex (depicted in Figure 6C and elaborated in Supplementary Note S2). The model incorporated a DNA binding step and a DNA cleavage step at the complex. We assumed the non-activated complex and the activated complex to share the same DNA binding kinetics, while exhibiting different DNA cleavage rates. We justified the equal binding kinetics, by the observation of a similar initial fast fluorescence increase. The reaction kinetics of the non-activated complex and the activated complex were assumed to follow a simple Michaelis–Menten scheme. Thus, the ssDNA substrate initially binds reversibly to Csm (rate constants $k_{{\mathrm{bind}}}^ +$ and $k_{{\mathrm{bind}}}^ - )$, and is subsequently cleaved and released in an irreversible step (rate constants $k_{{\mathrm{cleave}}}^N$ and $k_{{\mathrm{cleave}}}^A$, for both complexes, respectively). Releasing the cleaved DNA enables the complex to bind new DNA molecules. Furthermore, we introduced a mechanism to allow for the release of bound RNA by the activated complex, resulting in its deactivation with rate constant $k_{{\mathrm{RNA}}}^ -$. For simplicity, we assumed the RNA binding to be much faster than all other reaction steps in agreement with the previously observed rapid binding, cleavage, and turnover kinetics.

We next fitted a numerical solution of the set of differential rate equations (see Supplementary Note S2), that represent the model to the experimentally obtained fluorescence trajectories (see dashed lines in Figures 6A, B). The global fit successfully reproduced all aspects of the intricate time-dependent DNase activity of the Csm complex. Specifically, it accurately replicated the rapid initial increase, the DNA concentration-dependent slope of the background activity, as well as the amplitude and timescale of the decaying DNase activity in the presence of activating target RNA. From the fit, we obtained the rate constants of the simplified reaction scheme. For the rate constants representing the association and dissociation of the ssDNA to the Csm complex, we obtained values of $k_{{\mathrm{bind}}}^ + = ( {6.6 \pm 0.1} ) \cdot {10}^3$ /Ms and $k_{{\mathrm{bind}}}^ - = ( {1.1 \pm 0.1} ) \cdot {10}^{ - 2}$ /s, respectively. Of note, this represents a dissociation constant ${K}_d =$$k_{{\mathrm{bind}}}^ - /k_{{\mathrm{bind}}}^ +$ in the low micromolar range ($ \approx 2\ {\mathrm{\mu M}})$, which is higher than the highest ssDNA concentration in the measurements and should thus be considered as a rough estimate. Furthermore, for the rate constants describing the cleavage and release for the non-activated and the activated complex, we obtained values of $k_{{\mathrm{cleave}}}^N = ( {1.9 \pm 0.1} ) \cdot {10}^{ - 4}$ /s and $k_{{\mathrm{cleave}}}^A = ( {8.5 \pm 0.1} ) \cdot {10}^{ - 3}$ /s, respectively. Most importantly, the fit provided for the deactivation a rate constant $k_{{\mathrm{RNA}}}^ - = ( {1.9 \pm 0.1} ) \cdot {10}^{ - 4}$ /s, corresponding to a mean time of $\langle t \rangle = ( {89 \pm 1} )$ min. The agreement of this value with the values obtained before in this study additionally supports that this parameter can be attributed to the release of the RNA ends from the complex. Overall, we are confident that the model correctly captured the main steps of the DNase activity of the Csm complex, despite small deviations between the theoretical model and experimental data.

In conclusion, we thoroughly examined the kinetics of the Csm complex's DNase activity and demonstrated its ability to undergo multiple turnovers on ssDNA. Moreover, our findings indicated that RNA target recognition activates the complex, enhancing its ssDNA cleavage activity without affecting its DNA binding affinity. Lastly, we introduced and successfully parameterized a kinetic model that accurately captured these observations, enabling us to quantitatively describe the observed kinetics.

## Discussion and conclusion

Type III CRISPR-Cas systems are of particular interest due to their multifaceted defense mechanism against invading entities that acts in parallel at different levels. This intricate mechanism involves a coordinated orchestration of activities, encompassing RNA cleavage, DNA cleavage, and cA_*n*_ synthesis. To achieve orchestration, a spatiotemporal regulation mechanism has been proposed (7,20,23), by which target RNA binding turns on the Cas10 DNase and cA_*n*_ synthase activities which are subsequently switched off due to RNA cleavage by the Csm3 RNase. This is thought to provide a tight temporal control over DNA degradation and cA_*n*_ signaling. However, RNA cleavage can occur in seconds while the Csm complex activation can last up to hours (7), such that the precise temporal regulation of type III complex activation through RNA binding still remained enigmatic. In this study, we employed a combination of single-molecule and bulk-solution fluorescence experiments to carefully dissect the temporal regulation of RNA-activated DNA cleavage by a type III-A Csm complex from *Streptococcus thermophilus*.

First, we studied the duration at which RNA end fragments remain bound together at the Csm complex using dual-color FCS (Figure 2) and single-molecule TIRF microscopy (Supplementary Figure S5). This yielded a mean release time of the ends of $\langle t \rangle = ( {84 \pm 1} )$ min, which was in strong contrast to previously observed rapid RNA cleavage (7). Overall, this demonstrated that despite the RNA fragments were rapidly produced, the RNA ends remained bound to the complex for a much longer duration. Control experiments validated the specificity of RNA cleavage, the necessity of RNA cleavage for fragment release, and the independence of downstream activities of the RNA release (Supplementary Figure S4).

Evaluating the autocorrelation signal allowed us furthermore to probe the release of individual labeled RNA parts (Figures 3A–D). This provided similar release times for the Cas10-proximal and the Cas10-distal RNA end of $\langle t \rangle = ( {84 \pm 2} )$ min and $\langle t \rangle = ( {119 \pm 3} )$ min, respectively. Remarkably, as they are similar to the combined release time from the cross-correlation analysis, this suggests a coordinated release in which the release of one RNA end influences the release of the other. Given the shorter timescale of the Cas10-proximal end release, the release of the Cas10-proximal end may be accompanied by a conformational change within the complex, in turn facilitating the release of the Cas10-distal end (see below).

In addition to the RNA ends, we also probed the release of a short (6 nt) internal fragment that is produced upon RNA cleavage (Figures 3E, F). We obtained a bi-phasic decay of the mean diffusion time, wherein both phases decayed on a much shorter timescale (${\langle t \rangle }_1 = ( {1.0 \pm 0.1} )$ min and $\langle {{t}_2} \rangle = ( {25 \pm 4} )$ min) compared to the RNA end release. Overall, this poses a mechanism in which the RNA ends remain tethered to the complex on an hour timescale, while the short internal fragments are promptly released following the rapid cleavage process.

To understand the temporal control of the single-stranded DNA cleavage by the Cas10 subunit, we measured the DNase activity using continuous bulk-fluorescence experiments (Figure 4B). We confirmed that the DNase activity is triggered in a highly RNA-sequence specific manner and decayed over the timescale of several hours with a mean time of $\langle t \rangle = ( {68 \pm 1} )$ min. The timescale of the DNase activity agrees with the timescale of the RNA end binding, which provides evidence that the retention of the RNA ends acts as a molecular memory of the DNase activity. We speculate that the longer retention of the Cas10-proximal RNA cleavage product allows the complex to remain bound to the transcription bubble, ensuring close contact with the targeted ssDNA for efficient cleavage (50). Furthermore, we speculate that the retention of the RNA ends by the Csm complex will likely maintain the cA_*n*_ synthesis, too, as it decays on a similar timescale (20).

A requirement for bound RNA ends for the complex activation may also be exploited to discriminate between matching and mutated target sequences. To test this, we performed bulk fluorescence measurements employing substrates that form 9 base pair mismatches on either the 5′-end or the 3′-end of the crRNA (Supplementary Figure S8). No DNase activation beyond background level was detected which demonstrated that almost full target RNA binding is required for the Cas10 activation. In contrast, dual-color FCS measurements on the same substrates showed that despite the absence of DNase activity, the RNA ends remained bound to the complex for an elongated period. Thus, sole RNA end binding is not sufficient for Cas10 activation. This suggests an additional sequence discrimination mechanism, which e.g. communicates the presence of Cas10-distal mismatches to the Cas10 subunit despite internal fragment release. From structural data, it is known that inside Csm the crRNA lies deeply in a channel formed by the Csm2, Csm3, Csm5 and Cas10 subunits. Upon target RNA binding, the channel narrows and the protein subunits tightly surround the RNA duplex (37). It may be that in presence of sufficient mismatches at either end, the channel closure is perturbed and Cas10 remains in a non-activated state. Further investigations that allow probing the channel closure could shed light into the role of the channel closure. Notably, a major conformational change upon full target binding, activating downstream DNA cleavage, is known for the Cascade surveillance complex of Type I CRISPR-Cas systems (43,57–60). Beyond structural similarities (61,62,38), Cascade and Csm/Cmr complexes may thus also share mechanistic similarities, which however need to be investigated in more detail.

We also carefully investigated the turnover of target RNA on the Csm complex (Figures 4C and 5). We demonstrated that the DNase activity could be reactivated upon reintroducing target RNA at a later point. Consequently, we investigated a potential prolongation of the DNase activity, by providing an excess of activating target RNA. Interestingly, we found that higher RNA concentrations did not extend the duration of the DNase activity beyond a certain threshold, suggesting that the complex performs a rapid turnover of RNA strands while maintaining the duration of the DNase activity. This was supported by the observation that activated complex could be deactivated using non-activating RNA targets (Supplementary Figure S11), which can form 8 additional base pairs with the Cas10-proximal side, ensuring efficient self- and non-self-target discrimination during the timeframe of activity. A rapid RNA turnover could be directly observed on the RNA level by dual-color FCS measurements, demonstrating that excess RNA can displace bound targets. Based on our findings, we proposed a reaction scheme in which the Csm complex, upon RNA-target recognition and cleavage, initiates DNase activity and releases the short RNA fragments. The stretch of unpaired crRNA then facilitates a rapid binding of new RNA substrates while maintaining the DNase activity through bound RNA ends. This provides a rather constrained duration of the DNase activity of the complex, while ensuring efficient invader RNA degradation.

Lastly, we systematically investigated the ssDNA binding and cleavage dynamics of the Cas10 subunit by conducting further DNase activity measurements (Figure 6). Our measurements resolved an initial DNA binding step, followed by DNase activity that decayed on the same timescale over all tested ssDNA concentrations, suggesting that RNA end release provides an inherent decay mechanism independent of the ssDNA. We then constructed and parameterized a simplified kinetic model that successfully replicated all characteristics of the experimental data. We obtained an estimate for the dissociation constant ${K}_d$ of the binding of ssDNA to the Csm complex in the low micromolar regime. This low affinity may explain the challenges faced in obtaining crystal structures of the complex being bound to ssDNA. Remarkably, the obtained time constant of $\langle t \rangle = ( {89 \pm 1} )$ min for the deactivation of the complex agreed with the values obtained in other parts of the study, reinforcing its association with the RNA end release.

In conclusion, our study provides a thorough and comprehensive characterization of the mechanisms governing the RNA-dependent temporal control of the Csm DNase activity. We anticipate that our findings contribute significantly to a deeper understanding of the interwoven and multifaceted functions of the Csm complex, enhancing the general comprehension of its biological relevance and functions. Moreover, we expect our insights to provide valuable context for interpreting structural studies, connecting mechanistic insights with structural details.

## Supplementary Material

gkae080_Supplemental_File

## Data Availability

All presented data is available at: https://doi.org/10.5281/zenodo.10554510.

## References

[1] Nussenzweig P.M., Marraffini L.A. Molecular mechanisms of CRISPR-Cas immunity in bacteria. Annu. Rev. Genet. 2020; 54:93–120. 32857635 10.1146/annurev-genet-022120-112523

[2] Makarova K.S., Wolf Y.I., Iranzo J., Shmakov S.A., Alkhnbashi O.S., Brouns S.J.J., Charpentier E., Cheng D., Haft D.H., Horvath P. et al. Evolutionary classification of CRISPR-Cas systems: a burst of class 2 and derived variants. Nat. Rev. Microbiol. 2020; 18:67–83. 31857715 10.1038/s41579-019-0299-x PMC8905525

[3] Cui N., Zhang J.-T., Liu Y., Liu Y., Liu X.-Y., Wang C., Huang H., Jia N. Type IV-A CRISPR-csf complex: assembly, dsDNA targeting, and CasDinG recruitment. Mol. Cell. 2023; 83:2493–2508. 37343553 10.1016/j.molcel.2023.05.036

[4] Molina R., Sofos N., Montoya G. Structural basis of CRISPR-Cas Type III prokaryotic defence systems. Curr. Opin. Struct. Biol. 2020; 65:119–129. 32712502 10.1016/j.sbi.2020.06.010

[5] Tamulaitis G., Venclovas Č., Siksnys V. Type III CRISPR-Cas immunity: major differences brushed aside. Trends Microbiol. 2017; 25:49–61. 27773522 10.1016/j.tim.2016.09.012

[6] Samai P., Pyenson N., Jiang W., Goldberg G.W., Hatoum-Aslan A., Marraffini L.A. Co-transcriptional DNA and RNA cleavage during type III CRISPR-Cas immunity. Cell. 2015; 161:1164–1174. 25959775 10.1016/j.cell.2015.04.027 PMC4594840

[7] Kazlauskiene M., Tamulaitis G., Kostiuk G., Venclovas Č., Siksnys V. Spatiotemporal control of type III-A CRISPR-Cas immunity: coupling DNA degradation with the target RNA recognition. Mol. Cell. 2016; 62:295–306. 27105119 10.1016/j.molcel.2016.03.024

[8] Ramia N.F., Spilman M., Tang L., Shao Y., Elmore J., Hale C., Cocozaki A., Bhattacharya N., Terns R.M., Terns M.P. et al. Essential structural and functional roles of the Cmr4 subunit in RNA cleavage by the Cmr CRISPR-Cas complex. Cell Rep. 2014; 9:1610–1617. 25482566 10.1016/j.celrep.2014.11.007 PMC4269474

[9] Staals R.H.J., Agari Y., Maki-Yonekura S., Zhu Y., Taylor D.W., van Duijn E., Barendregt A., Vlot M., Koehorst J.J., Sakamoto K. et al. Structure and activity of the RNA-targeting type III-B CRISPR-Cas complex of Thermus thermophilus. Mol. Cell. 2013; 52:135–145. 24119403 10.1016/j.molcel.2013.09.013 PMC4006948

[10] Tamulaitis G., Kazlauskiene M., Manakova E., Venclovas Č., Nwokeoji A.O., Dickman M.J., Horvath P., Siksnys V. Programmable RNA shredding by the type III-A CRISPR-Cas system of *Streptococcus thermophilus*. Mol. Cell. 2014; 56:506–517. 25458845 10.1016/j.molcel.2014.09.027

[11] Staals R.H.J., Zhu Y., Taylor D.W., Kornfeld J.E., Sharma K., Barendregt A., Koehorst J.J., Vlot M., Neupane N., Varossieau K. et al. RNA targeting by the type III-A CRISPR-Cas csm complex of Thermus thermophilus. Mol. Cell. 2014; 56:518–530. 25457165 10.1016/j.molcel.2014.10.005 PMC4342149

[12] Elmore J.R., Sheppard N.F., Ramia N., Deighan T., Li H., Terns R.M., Terns M.P. Bipartite recognition of target RNAs activates DNA cleavage by the type III-B CRISPR-Cas system. Genes Dev. 2016; 30:447–459. 26848045 10.1101/gad.272153.115 PMC4762429

[13] Estrella M.A., Kuo F.-T., Bailey S. RNA-activated DNA cleavage by the type III-B CRISPR-Cas effector complex. Genes Dev. 2016; 30:460–470. 26848046 10.1101/gad.273722.115 PMC4762430

[14] Han W., Li Y., Deng L., Feng M., Peng W., Hallstrøm S., Zhang J., Peng N., Liang Y.X., White M.F. et al. A type III-B CRISPR-Cas effector complex mediating massive target DNA destruction. Nucleic Acids Res. 2017; 45:1983–1993. 27986854 10.1093/nar/gkw1274 PMC5389615

[15] Liu T.Y., Iavarone A.T., Doudna J.A. Correction: RNA and DNA targeting by a reconstituted thermus thermophilus type III-A CRISPR-Cas system. PLoS One. 2017; 12:e0175612. 28114398 10.1371/journal.pone.0170552 PMC5256923

[16] Lin J., Feng M., Zhang H., She Q. Characterization of a novel type III CRISPR-Cas effector provides new insights into the allosteric activation and suppression of the Cas10 DNase. Cell Discov. 2020; 6:29. 32411384 10.1038/s41421-020-0160-4 PMC7214462

[17] Lin J., Shen Y., Ni J., She Q. A type III-A CRISPR-Cas system mediates co-transcriptional DNA cleavage at the transcriptional bubbles in close proximity to active effectors. Nucleic Acids Res. 2021; 49:7628–7643. 34197611 10.1093/nar/gkab590 PMC8287949

[18] Liu T.Y., Liu J.-J., Aditham A.J., Nogales E., Doudna J.A. Target preference of type III-A CRISPR-Cas complexes at the transcription bubble. Nat. Commun. 2019; 10:3001. 31278272 10.1038/s41467-019-10780-2 PMC6611850

[19] Mo C.Y., Mathai J., Rostøl J.T., Varble A., Banh D.V., Marraffini L.A. Type III-A CRISPR immunity promotes mutagenesis of staphylococci. Nature. 2021; 592:611–615. 33828299 10.1038/s41586-021-03440-3 PMC8820005

[20] Kazlauskiene M., Kostiuk G., Venclovas Č., Tamulaitis G., Siksnys V. A cyclic oligonucleotide signaling pathway in type III CRISPR-Cas systems. Science. 2017; 357:605–609. 28663439 10.1126/science.aao0100

[21] Niewoehner O., Garcia-Doval C., Rostøl J.T., Berk C., Schwede F., Bigler L., Hall J., Marraffini L.A., Jinek M. Type III CRISPR-Cas systems produce cyclic oligoadenylate second messengers. Nature. 2017; 548:543–548. 28722012 10.1038/nature23467

[22] Han W., Stella S., Zhang Y., Guo T., Sulek K., Peng-Lundgren L., Montoya G., She Q. A type III-B cmr effector complex catalyzes the synthesis of cyclic oligoadenylate second messengers by cooperative substrate binding. Nucleic Acids Res. 2018; 46:10319–10330. 30239876 10.1093/nar/gky844 PMC6212834

[23] Rouillon C., Athukoralage J.S., Graham S., Grüschow S., White M.F. Control of cyclic oligoadenylate synthesis in a type III CRISPR system. eLife. 2018; 7:e36734. 29963983 10.7554/eLife.36734 PMC6053304

[24] Grüschow S., Athukoralage J.S., Graham S., Hoogeboom T., White M.F. Cyclic oligoadenylate signalling mediates mycobacterium tuberculosis CRISPR defence. Nucleic Acids Res. 2019; 47:9259–9270. 31392987 10.1093/nar/gkz676 PMC6755085

[25] Nasef M., Muffly M.C., Beckman A.B., Rowe S.J., Walker F.C., Hatoum-Aslan A., Dunkle J.A. Regulation of cyclic oligoadenylate synthesis by the Staphylococcus epidermidis Cas10-csm complex. RNA. 2019; 25:948–962. 31076459 10.1261/rna.070417.119 PMC6633199

[26] Smalakyte D., Kazlauskiene M., F Havelund J., Rukšėnaitė A., Rimaite A., Tamulaitiene G., Færgeman N.J., Tamulaitis G., Siksnys V Type III-A CRISPR-associated protein Csm6 degrades cyclic hexa-adenylate activator using both CARF and HEPN domains. Nucleic Acids Res. 2020; 48:9204–9217. 32766806 10.1093/nar/gkaa634 PMC7498309

[27] Foster K., Grüschow S., Bailey S., White M.F., Terns M.P. Regulation of the RNA and DNA nuclease activities required for pyrococcus furiosus type III-B CRISPR-Cas immunity. Nucleic Acids Res. 2020; 48:4418–4434. 32198888 10.1093/nar/gkaa176 PMC7192623

[28] McMahon S.A., Zhu W., Graham S., Rambo R., White M.F., Gloster T.M. Structure and mechanism of a type III CRISPR defence DNA nuclease activated by cyclic oligoadenylate. Nat. Commun. 2020; 11:500. 31980625 10.1038/s41467-019-14222-x PMC6981274

[29] Rostøl J.T., Xie W., Kuryavyi V., Maguin P., Kao K., Froom R., Patel D.J., Marraffini L.A. The Card1 nuclease provides defence during type III CRISPR immunity. Nature. 2021; 590:624–629. 33461211 10.1038/s41586-021-03206-x PMC7906951

[30] Zhu W., McQuarrie S., Grüschow S., McMahon S.A., Graham S., Gloster T.M., White M.F. The CRISPR ancillary effector Can2 is a dual-specificity nuclease potentiating type III CRISPR defence. Nucleic Acids Res. 2021; 49:2777–2789. 33590098 10.1093/nar/gkab073 PMC7969007

[31] Rouillon C., Schneberger N., Chi H., Blumenstock K., Da Vela S., Ackermann K., Moecking J., Peter M.F., Boenigk W., Seifert R. et al. Antiviral signalling by a cyclic nucleotide activated CRISPR protease. Nature. 2023; 614:168–174. 36423657 10.1038/s41586-022-05571-7

[32] Charbonneau A.A., Eckert D.M., Gauvin C.C., Lintner N.G., Lawrence C.M. Cyclic tetra-adenylate (cA4) recognition by Csa3; implications for an integrated class 1 CRISPR-Cas Iimmune response in Saccharolobus solfataricus. Biomolecules. 2021; 11:1852. 34944496 10.3390/biom11121852 PMC8699464

[33] Mogila I., Tamulaitiene G., Keda K., Timinskas A., Ruksenaite A., Sasnauskas G., Venclovas Č., Siksnys V., Tamulaitis G. Ribosomal stalk-captured CARF-RelE ribonuclease inhibits translation following CRISPR signaling. Science. 2023; 382:1036–1041. 38033086 10.1126/science.adj2107

[34] Jiang W., Samai P., Marraffini L.A. Degradation of phage transcripts by CRISPR-associated RNases enables type III CRISPR-Cas immunity. Cell. 2016; 164:710–721. 26853474 10.1016/j.cell.2015.12.053 PMC4752873

[35] Rostøl J.T., Marraffini L.A. Non-specific degradation of transcripts promotes plasmid clearance during type III-A CRISPR-Cas immunity. Nat. Microbiol. 2019; 4:656–662. 30692669 10.1038/s41564-018-0353-x PMC6430669

[36] Foster K., Kalter J., Woodside W., Terns R.M., Terns M.P. The ribonuclease activity of Csm6 is required for anti-plasmid immunity by Type III-A CRISPR-Cas systems. RNA Biology. 2019; 16:449–460. 29995577 10.1080/15476286.2018.1493334 PMC6546353

[37] You L., Ma J., Wang J., Artamonova D., Wang M., Liu L., Xiang H., Severinov K., Zhang X., Wang Y. Structure studies of the CRISPR-Csm complex reveal mechanism of Co-transcriptional interference. Cell. 2019; 176:239–253. 30503210 10.1016/j.cell.2018.10.052 PMC6935017

[38] Jia N., Mo C.Y., Wang C., Eng E.T., Marraffini L.A., Patel D.J. Type III-A CRISPR-Cas csm complexes: assembly, periodic RNA cleavage, DNase activity regulation, and autoimmunity. Mol. Cell. 2019; 73:264–277. 30503773 10.1016/j.molcel.2018.11.007 PMC6355164

[39] Sofos N., Feng M., Stella S., Pape T., Fuglsang A., Lin J., Huang Q., Li Y., She Q., Montoya G. Structures of the cmr-β complex reveal the regulation of the immunity mechanism of type III-B CRISPR-Cas. Mol. Cell. 2020; 79:741–757. 32730741 10.1016/j.molcel.2020.07.008

[40] Sternberg S.H., Redding S., Jinek M., Greene E.C., Doudna J.A. DNA interrogation by the CRISPR RNA-guided endonuclease Cas9. Nature. 2014; 507:62–67. 24476820 10.1038/nature13011 PMC4106473

[41] Szczelkun M.D., Tikhomirova M.S., Sinkunas T., Gasiunas G., Karvelis T., Pschera P., Siksnys V., Seidel R. Direct observation of R-loop formation by single RNA-guided Cas9 and Cascade effector complexes. Proc. Nat. Acad. Sci. U.S.A. 2014; 111:9798–9803. 10.1073/pnas.1402597111 PMC4103346 24912165

[42] Redding S., Sternberg S.H., Marshall M., Gibb B., Bhat P., Guegler C.K., Wiedenheft B., Doudna J.A., Greene E.C. Surveillance and processing of foreign DNA by the Escherichia coli CRISPR-Cas system. Cell. 2015; 163:854–865. 26522594 10.1016/j.cell.2015.10.003 PMC4636941

[43] Rutkauskas M., Sinkunas T., Songailiene I., Tikhomirova M.S., Siksnys V., Seidel R. Directional R-loop formation by the CRISPR-Cas surveillance complex cascade provides efficient off-target site rejection. Cell Rep. 2015; 10:1534–1543. 25753419 10.1016/j.celrep.2015.01.067

[44] Sternberg S.H., LaFrance B., Kaplan M., Doudna J.A. Conformational control of DNA target cleavage by CRISPR-Cas9. Nature. 2015; 527:110–113. 26524520 10.1038/nature15544 PMC4859810

[45] Xue C., Whitis N.R., Sashital D.G. Conformational control of cascade interference and priming activities in CRISPR immunity. Mol. Cell. 2016; 64:826–834. 27871367 10.1016/j.molcel.2016.09.033 PMC5561731

[46] Dillard K.E., Brown M.W., Johnson N.V., Xiao Y., Dolan A., Hernandez E., Dahlhauser S.D., Kim Y., Myler L.R., Anslyn E.V. et al. Assembly and translocation of a CRISPR-Cas primed acquisition complex. Cell. 2018; 175:934–946. 30343903 10.1016/j.cell.2018.09.039 PMC6441324

[47] Loeff L., Brouns S.J.J., Joo C. Repetitive DNA reeling by the cascade-Cas3 complex in nucleotide unwinding steps. Mol. Cell. 2018; 70:385–394. 29706536 10.1016/j.molcel.2018.03.031

[48] Singh D., Mallon J., Poddar A., Wang Y., Tippana R., Yang O., Bailey S., Ha T. Real-time observation of DNA target interrogation and product release by the RNA-guided endonuclease CRISPR Cpf1 (Cas12a). Proc. Nat. Acad. Sci. U.S.A. 2018; 115:5444–5449. 10.1073/pnas.1718686115 PMC6003496 29735714

[49] Wang L., Mo C.Y., Wasserman M.R., Rostøl J.T., Marraffini L.A., Liu S. Dynamics of Cas10 govern discrimination between self and non-self in type III CRISPR-Cas immunity. Mol. Cell. 2019; 73:278–290. 30503774 10.1016/j.molcel.2018.11.008 PMC6338483

[50] Mogila I., Kazlauskiene M., Valinskyte S., Tamulaitiene G., Tamulaitis G., Siksnys V. Genetic dissection of the type III-A CRISPR-Cas system csm complex reveals roles of individual subunits. Cell Rep. 2019; 26:2753–2765. 30840895 10.1016/j.celrep.2019.02.029

[51] Schrimpf W., Barth A., Hendrix J., Lamb D.C. PAM: a framework for Integrated analysis of imaging, single-molecule, and ensemble fluorescence data. Biophys. J. 2018; 114:1518–1528. 29642023 10.1016/j.bpj.2018.02.035 PMC5954487

[52] Harris C.R., Millman K.J., van der Walt S.J., Gommers R., Virtanen P., Cournapeau D., Wieser E., Taylor J., Berg S., Smith N.J. et al. Array programming with NumPy. Nature. 2020; 585:357–362. 32939066 10.1038/s41586-020-2649-2 PMC7759461

[53] Laine R.F., Tosheva K.L., Gustafsson N., Gray R.D.M., Almada P., Albrecht D., Risa G.T., Hurtig F., Lindås A.-C., Baum B. et al. NanoJ: a high-performance open-source super-resolution microscopy toolbox. J. Phys. D Appl. Phys. 2019; 52:163001. 33191949 10.1088/1361-6463/ab0261 PMC7655149

[54] Müller B.K., Zaychikov E., Bräuchle C., Lamb D.C. Pulsed interleaved excitation. Biophys. J. 2005; 89:3508–3522. 16113120 10.1529/biophysj.105.064766 PMC1366845

[55] Lakowicz J.R. Principles of Fluorescence Spectroscopy. 2006; 3rd edn. NY Springer.

[56] Kettling U., Koltermann A., Schwille P., Eigen M. Real-time enzyme kinetics monitored by dual-color fluorescence cross-correlation spectroscopy. Proc. Nat. Acad. Sci. U.S.A. 1998; 95:1416–1420. 10.1073/pnas.95.4.1416 PMC19026 9465029

[57] Rutkauskas M., Songailiene I., Irmisch P., Kemmerich F.E., Sinkunas T., Siksnys V., Seidel R. A quantitative model for the dynamics of target recognition and off-target rejection by the CRISPR-Cas Cascade complex. Nat. Commun. 2022; 13:7460. 36460652 10.1038/s41467-022-35116-5 PMC9718816

[58] Kauert D.J., Madariaga-Marcos J., Rutkauskas M., Wulfken A., Songailiene I., Sinkunas T., Siksnys V., Seidel R. The energy landscape for R-loop formation by the CRISPR-Cas Cascade complex. Nat. Struct. Mol. Biol. 2023; 30:1040–1047. 37415009 10.1038/s41594-023-01019-2

[59] Xiao Y., Luo M., Dolan A.E., Liao M., Ke A. Structure basis for RNA-guided DNA degradation by Cascade and Cas3. Science. 2018; 361:eaat0839. 29880725 10.1126/science.aat0839 PMC6537108

[60] Xiao Y., Luo M., Hayes R.P., Kim J., Ng S., Ding F., Liao M., Ke A. Structure basis for directional R-loop formation and substrate handover mechanisms in type I CRISPR-Cas system. Cell. 2017; 170:48–60. 28666122 10.1016/j.cell.2017.06.012 PMC5841471

[61] Mulepati S., Héroux A., Bailey S. Structural biology. Crystal structure of a CRISPR RNA-guided surveillance complex bound to a ssDNA target. Science. 2014; 345:1479–1484. 25123481 10.1126/science.1256996 PMC4427192

[62] Taylor D.W., Zhu Y., Staals R.H.J., Kornfeld J.E., Shinkai A., van der Oost J., Nogales E., Doudna J.A. Structural biology. Structures of the CRISPR-Cmr complex reveal mode of RNA target positioning. Science. 2015; 348:581–585. 25837515 10.1126/science.aaa4535 PMC4582657

