# Supplementary material for: Retention of the RNA ends provides the molecular memory for maintaining the activation of the Csm complex
Source: Nucleic Acids Res. 2024 Feb 10;52(7):3896–910. doi: 10.1093/nar/gkae080 (PMC11040160; doi:10.1093/nar/gkae080)
Supplement: gkae080_Supplemental_File [file gkae080_supplemental_file.pdf]

Supplementary Information

Retention of the RNA ends provides the molecular memory for maintaining the activation of the Csm complex

Patrick Irmisch[1], Irmantas Mogila[2], Brighton Samatanga[1], Gintautas Tamulaitis[2,*], and Ralf Seidel[1,*]

[1] Peter Debye Institute for Soft Matter Physics, University of Leipzig, Leipzig, 04103, Germany
[2] Institute of Biotechnology, Life Sciences Center, Vilnius University, Vilnius 10257, Lithuania

* To whom correspondence should be addressed. Tel: +49 341 9732501; Email: ralf.seidel@physik.uni-leipzig.de
Correspondence may also be addressed to Email: gintautas.tamulaitis@bti.vu.lt

Note S1: Models describing diffusion through the confocal volume in Fluorescence Correlation spectroscopy measurements.

To describe the measured fluorescence correlation curves, we applied models based on the correlation function for diffusion in three dimensions and a detection based on a Gaussian profile (1).

For the fluorescence cross-correlation in the dual-color experiments, a simple 3D-diffusion model was applied to describe the correlation signal G depending on the lag time τ:

$$G(\tau) = G_0 \frac{1}{\left(1+\frac{\tau}{\tau_D}\right)} \frac{1}{\sqrt{1+\frac{\tau}{k^2\tau_D}}},$$

where G_0 is the correlation amplitude, τ_D the diffusion time and $k = 4.9$ (as obtained by the manufacturer) an instrument specific constant describing the vertical stretching of the confocal volume compare to a sphere.

The red autocorrelation was fitted using a model including 2 diffusing species (having equal brightness) with separated amplitudes (G_1, G_2) and diffusion times (τ_1, τ_2):

$$G(\tau) = G_1 \frac{1}{\left(1+\frac{\tau}{\tau_1}\right)} \frac{1}{\sqrt{1+\frac{\tau}{k^2\tau_1}}} + G_2 \frac{1}{\left(1+\frac{\tau}{\tau_2}\right)} \frac{1}{\sqrt{1+\frac{\tau}{k^2\tau_2}}}$$

For the green autocorrelation, a significant portion of dyes being excited to the triplet state need to be accounted for, thus the model is expanded by an additional amplitude T and triplet-state lifetime τ_T:

$$G(\tau) = \left(G_1 \frac{1}{\left(1+\frac{\tau}{\tau_1}\right)} \frac{1}{\sqrt{1+\frac{\tau}{k^2\tau_1}}} + G_2 \frac{1}{\left(1+\frac{\tau}{\tau_2}\right)} \frac{1}{\sqrt{1+\frac{\tau}{k^2\tau_2}}} \right) \left(1 + \frac{T}{1-T} e^{-\frac{\tau}{\tau_T}}\right)$$

In case of two species, the mean diffusion time was determined by:

$$\tau_D = \frac{G_1\tau_1 + G_2\tau_2}{G_1 + G_2}$$

Note S2: Kinetic model for the RNA-dependent ssDNA cleavage by the Csm complex

The bulk fluorescence data obtained for the ssDNA-concentration dependence of the Csm DNase activity on ssDNA (Figure 6A, B) was modeled following a kinetic reaction scheme similar to Figure 6C.

In principle, we assumed the non-activated complex (not bound to any RNA fragments) and the activated complex (bound to RNA fragments) to share the same single-stranded DNA binding kinetics, while differing in DNA cleavage.

The reaction kinetics of the non-activated complex (*N)* and the activated complex (*A)* were assumed to follow a simple Michaelis-Menten scheme. In this scheme, the single stranded DNA substrate (*S)* initially binds to the complex in a reversible step and is subsequently cleaved and released in an irreversible step, yielding the product (*P).* Releasing the product enables the complex to bind new substrate molecules. This scheme is represented by the following relations:

$$N + S \underset{k_{\text{bind}}^{-}}{\overset{k_{\text{bind}}^{+}}{\rightleftharpoons}} NS \xrightarrow{k_{\text{cleave}}^{N}} N + P, \tag{1}$$

$$A + S \underset{k_{\text{bind}}^{-}}{\overset{k_{\text{bind}}^{+}}{\rightleftharpoons}} AS \xrightarrow{k_{\text{cleave}}^{A}} A + P, \tag{2}$$

with k_{bind}^{+} being a second order rate constant, and k_{bind}^{-}, k_{cleave}^{N} and k_{cleave}^{A} being first order rate constants. Notably, the rate constants describing the binding were assumed equal for both complex entities.

Additionally, we incorporated a mechanism allowing the activated complex to release bound RNA, leading to its deactivation according to:

$$A \xrightarrow{k_{\text{RNA}}^{-}} N, \tag{3}$$

$$AS \xrightarrow{k_{\text{RNA}}^{-}} NS, \tag{4}$$

with k_{RNA}^{-} being a first order rate constant. For simplicity, we chose to omit the consideration of RNA binding in our model. This simplification is justified by our earlier findings that indicated that both RNA binding and cleavage occur rapidly in comparison to the DNase activity of the complex. Moreover, the experiments were conducted at an equimolar ratio of Csm to RNA, ensuring that RNA turnover should not take place.

The set of differential equations describing the RNA-dependent DNase activity of the Csm complex is then given by:

$$\frac{d[N]}{dt} = -k_{\text{bind}}^{+}[N][S] + k_{\text{bind}}^{-}[NS] + k_{\text{cleave}}^{N}[NS] + k_{\text{RNA}}^{-}[N], \quad (5)$$

$$\frac{d[A]}{dt} = -k_{\text{bind}}^{+}[A][S] + k_{\text{bind}}^{-}[AS] + k_{\text{cleave}}^{A}[AS] - k_{\text{RNA}}^{-}[A], \quad (6)$$

$$\frac{d[S]}{dt} = -k_{\text{bind}}^{+}[N][S] - k_{\text{bind}}^{+}[A][S] + k_{\text{bind}}^{-}[NS] + k_{\text{bind}}^{-}[AS], \quad (7)$$

$$\frac{d[NS]}{dt} = +k_{\text{bind}}^{+}[N][S] - k_{\text{bind}}^{-}[NS] - k_{\text{cleave}}^{N}[NS] + k_{\text{RNA}}^{-}[AS], \quad (8)$$

$$\frac{d[AS]}{dt} = +k_{\text{bind}}^{+}[A][S] - k_{\text{bind}}^{-}[AS] - k_{\text{cleave}}^{A}[AS] - k_{\text{RNA}}^{-}[AS], \quad (9)$$

$$\frac{d[P]}{dt} = +k_{\text{cleave}}^{N}[NS] + k_{\text{cleave}}^{A}[AS]. \quad (10)$$

The obtained time course of cleaved ssDNA product (*P*) was related to the measured fluorescence signal representing cleaved ssDNA.

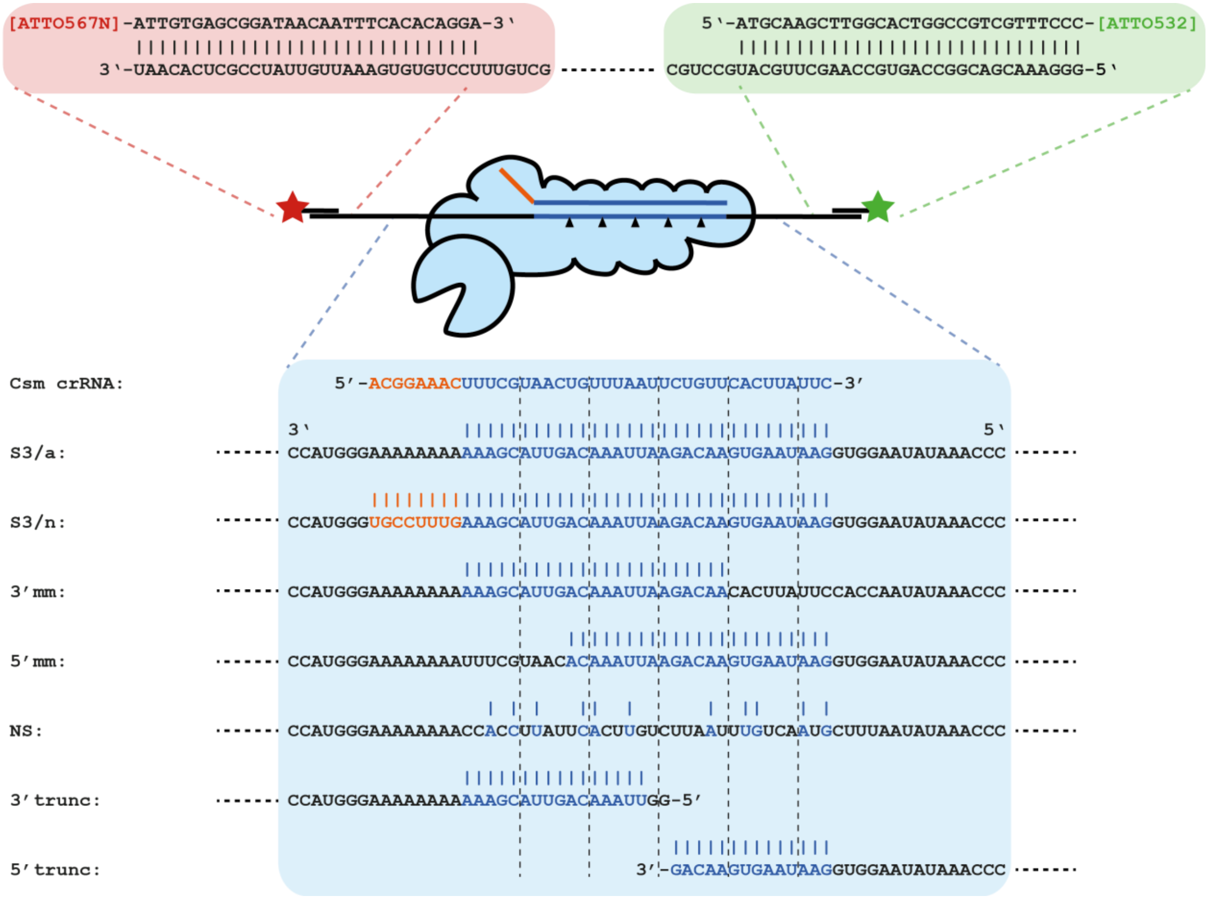

Figure S1: Scheme of the RNA substrates used for the FCS measurements. Full RNA and DNA sequences are summarized in Tables S1 and S2. Cleavage positions are indicated as dashed lines.

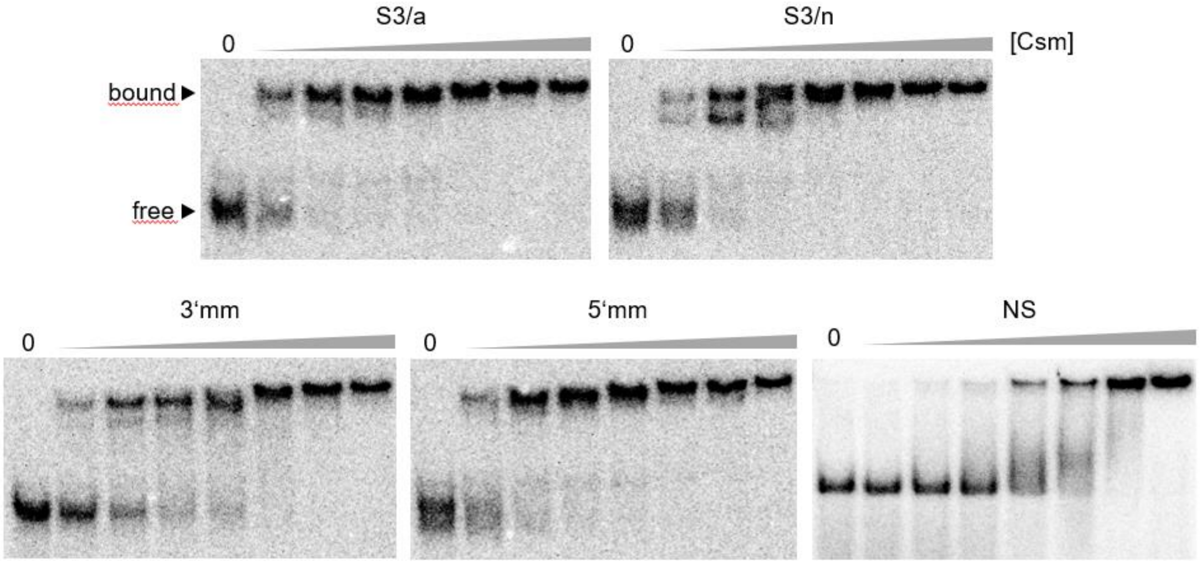

Figure S2: RNA binding by Csm. 50 pM of 5'-radiolabeled RNA substrates were incubated with 0, 0.1, 0.3, 1, 3, 10, 30, 100 nM of Csm, and RNA binding was assessed by electrophoretic mobility shift assay. The substrates used in each assay are denoted above the radiograms.

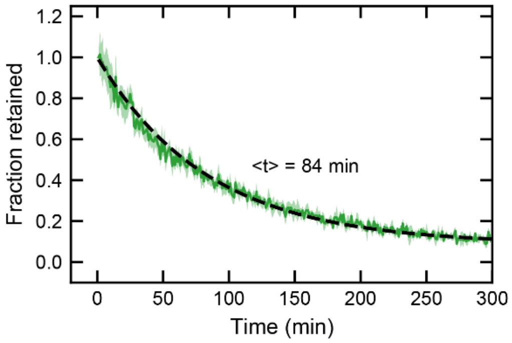

Figure S3: Characteristic timescale fit of RNA end release obtained from dual-color FCS measurements. The retained fraction of RNA ends (solid green line) was obtained by dividing the mean trace in presence of Csm by the mean trace in absence of Csm complex (see Figure 2C of the main text). Colored areas indicate an error on each data point obtained from the standard deviation of the triplicate measurement and using the error propagation formula. An exponential fit (dashed black line) to the experimental data provided a characteristic timescale of $\langle t \rangle = (84 \pm 1)$ min and a residual amplitude of $R_0 = (8.7 \pm 0.2)$ %.

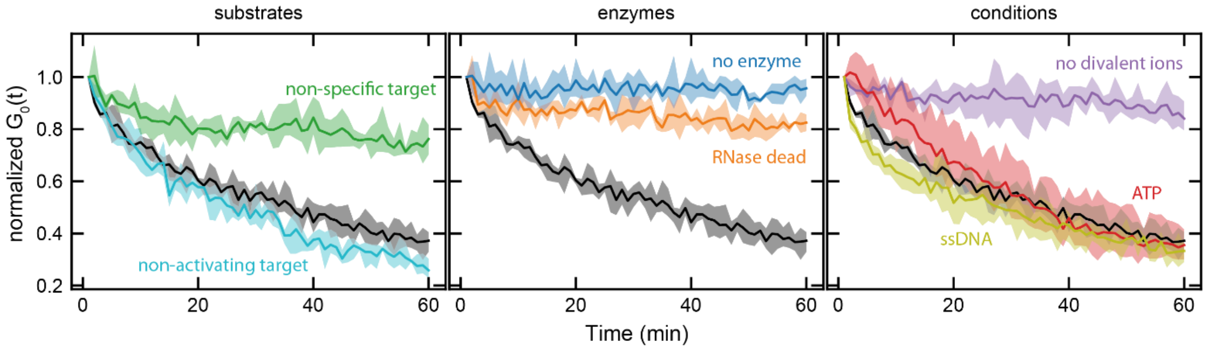

Figure S4: Fluorescence cross-correlation spectroscopy control experiments. Measured time dependent cross-correlation amplitudes for experiment alterations compared to the standard case (shown in black with 2 nM activating target RNA S3/a, 10 nM Csm and 0.5 mM $MnCl_2$). See Table S1 for used non-specific NS and non-activating S3/n RNA substrates. Solid lines indicate the mean of the signals for three repeated measurements, while colored areas indicate the range between minimum and maximum measured values at each time point. All traces were normalized against the first data point. The measurements involving ATP and ssDNA were conducted using concentrations of 5 mM ATP and 100 nM unlabeled ssDNA (Table S2).

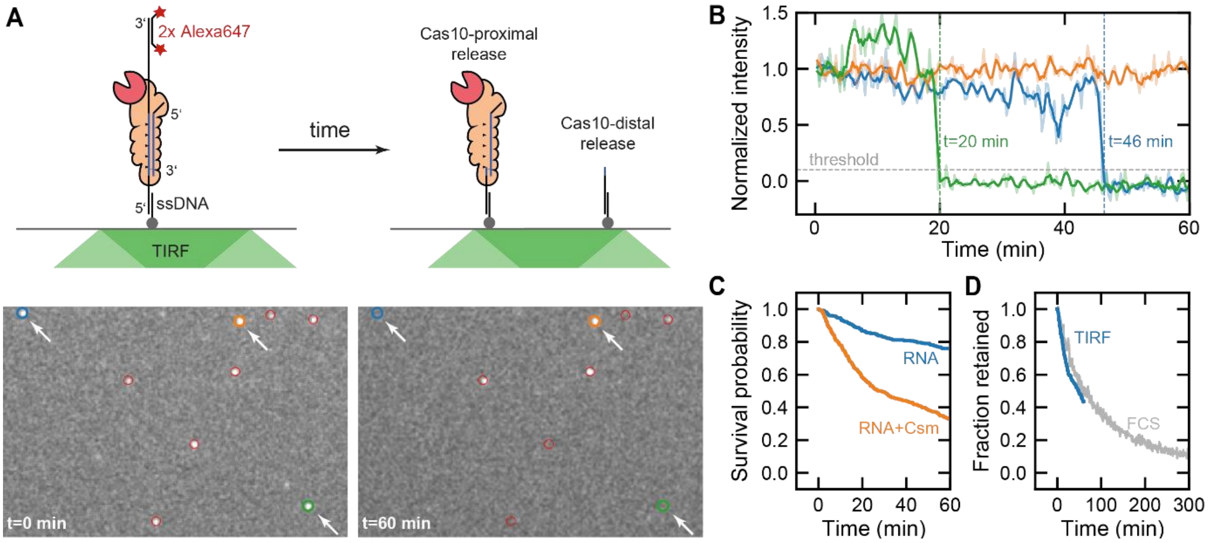

Figure S5: Following RNA end release by the Csm complex using total internal reflection fluorescence microscopy (TIRF). (**A**) Scheme of the TIRF experiment, with the Csm complex bound to the activating target RNA S3/a that is immobilized at its 5'-end and labeled with Alexa647 on its 3'-end. Fluorescence imaging allows following the release of the RNA fragments over time as shown in two example images at the bottom. Red circles in the images represent spots of single RNA molecules, identified using a spot tracking algorithm, some of which disappear over time. For the experiment, 2 nM of the immobilized activating target RNA S3/a was incubated with 10 nM of Csm in the absence of divalent ions. The reaction was initiated by adding divalent ions. (**B**) Normalized fluorescence intensities of the spots highlighted in (A) as a function of time, along with the 3-point moving average. Release of the Cas10-proximal or Cas10-distal RNA end results in a sudden drop in the fluorescence signal. A spot was considered disappeared when the moving average of the intensity dropped below 10% of its initial value. (**C**) Survival probability over time for measurements conducted in the absence (blue) and presence (orange) of Csm. The survival probability was determined by tracking the fluorescence intensities of N=504 and N=513 molecules in the absence and presence of Csm, respectively. (**D**) Fraction of retained molecules over time (blue line), calculated by dividing the survival probability in the presence of Csm by the survival probability in its absence. Additionally shown is the fraction of retained molecules obtained from dual-color FCS measurements (gray line, compare Figure S3).

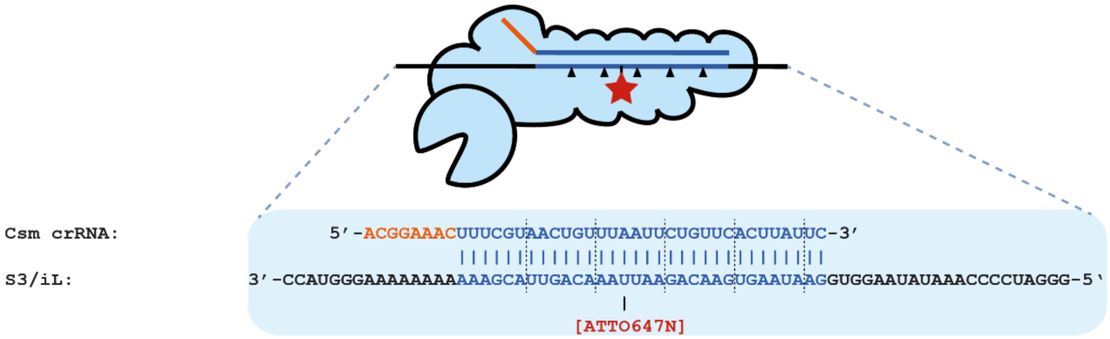

Figure S6: Scheme of the internally labeled substrate used for the FCS measurements. Sequence is listed in Table S1. Cleavage positions are indicated as dashed lines.

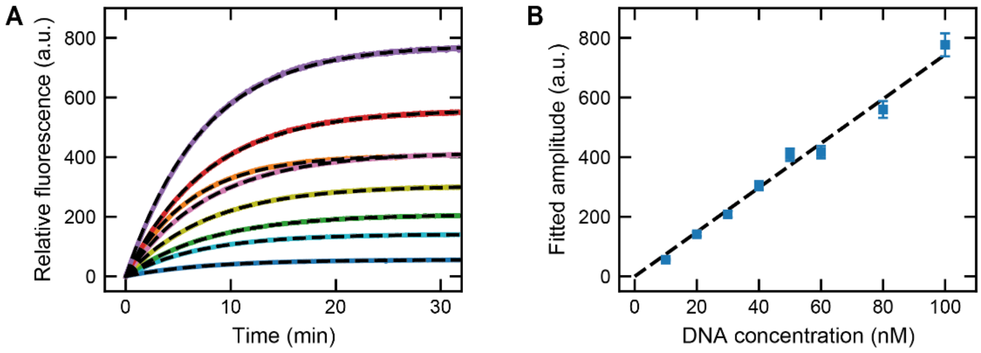

Figure S7: DNA cleavage experiment used to relate fluorescence intensities to cleaved single-stranded DNA. (**A**) Measured fluorescence traces (solid lines) together with an exponential fit (dashed lines) for increasing concentrations of DNA. (**B**) Fitted amplitudes (solid scatter points) obtained in (A) in dependence of the DNA concentration together with a linear fit (dashed line). The linear fit provided a linear scaling factor of 7.44 fluorescence units = 1 nM cleaved DNA. Measurements were performed employing 0.5 mM $MnCl_2$, 10 U/µl micrococcal nuclease and single-stranded DNA concentrations of 10, 20, 30, 40, 50, 60, 80 and 100 nM.

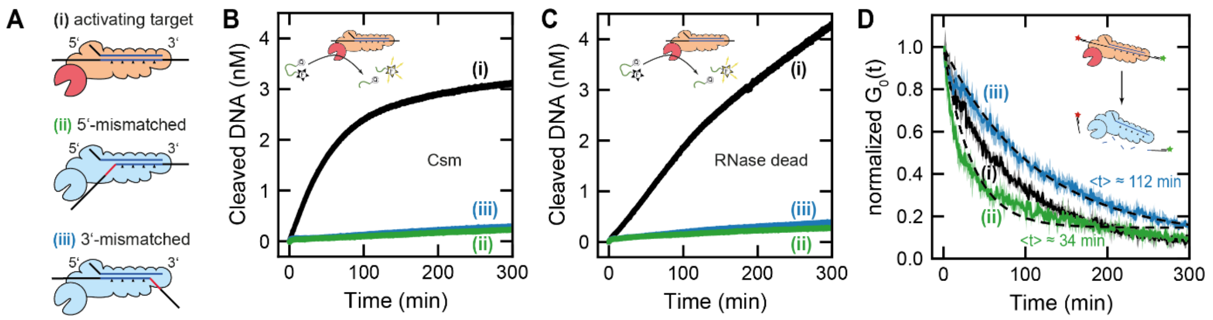

Figure S8: Bulk fluorescence and dual-color FCS measurements employing mismatched RNA substrates. (A) Schemes depicting the activating-target RNA (**i**), a 5'-mismatched-target RNA (**ii**), and a 3'-mismatched target RNA (**iii**), being bound to the Csm complex. (**B**) Time dependent bulk fluorescence measurements of the 3 substrates shown in (A), employing 2 nM of the respective RNA, 2 nM Csm and 100 nM double-labeled ssDNA. (**C**) Time dependent bulk fluorescence measurements of the 3 substrates shown in (A) (see also Table S1), employing 2 nM of the respective RNA, 2 nM of the Csm RNase dead mutant (D33A) and 100 nM double-labeled ssDNA. (**D**) Time dependent cross-correlation amplitude normalized by the first data point for measurements of the 3 substrates shown in (A), employing 10 nM of Csm and 2 nM of the respective RNA, hybridized to an oligonucleotide carrying a red label (ATTO-647N) and to another carrying a green label (ATTO-532). Exponential fits to the data (dashed lines) provided a mean RNA end release time for the 5'-mismatched substrate of $\langle t \rangle = (34 \pm 1)$ min and for the 3'-mismatched substrate of $\langle t \rangle = (112 \pm 1)$ min. Solid lines represent the mean of three repeated measurements, while colored areas in (D) represent the minimum and maximum values.

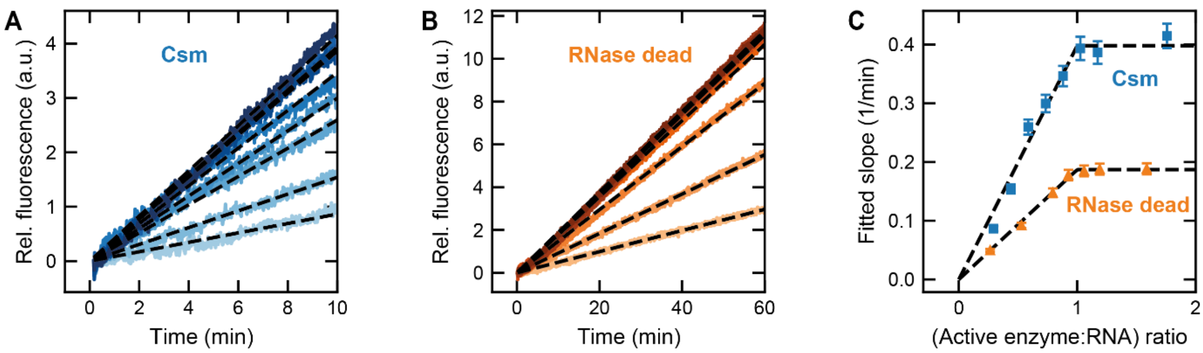

Figure S9: Determination of the active enzyme concentration from steady state kinetics. Fluorescence trajectories of the steady state cleavage (solid lines), employing 100nM ssDNA, 2 nM activating target RNA S3/a, and 1, 1.5, 2, 2.5, 3, 3.5, 4, 6 nM of Csm (**A**) and 1, 2, 3, 3.5, 4, 4.5, 6 nM of the Csm RNase-dead mutant (D33A) (**B**), together with a linear fit (dashed lines). (**C**) Slopes obtained from the fits in (A) and (B) together with a fit to stoichiometric behavior (high RNA affinity) provides the active enzyme concentration.

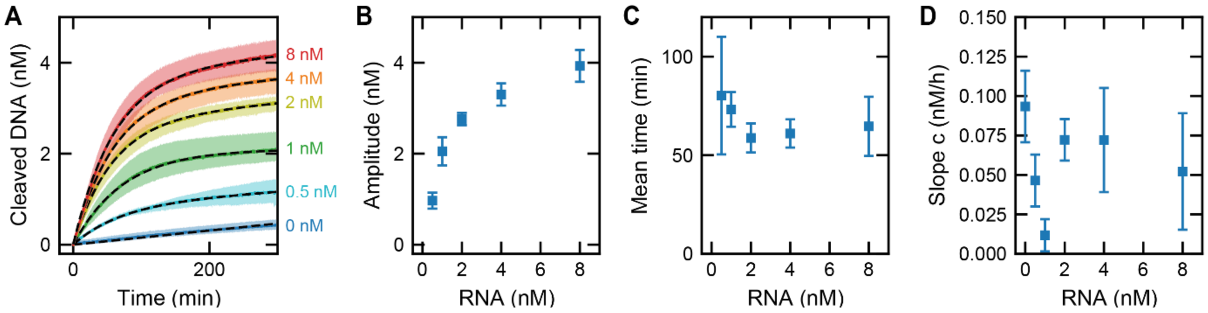

Figure S10: Exponential fitting of the RNA turnover kinetics. (**A**) Fluorescence traces (solid lines) together with an exponential fit (dashed lines). The traces represent the mean and colored areas indicate maximum and minimum values of three measurement repeats. The fit provided the amplitude (**B**), the mean time of the reaction (**C**) and the slope (background activity) (**D**). Error bars represent the standard deviation between the best-fit parameters obtained by the three measurement repeats.

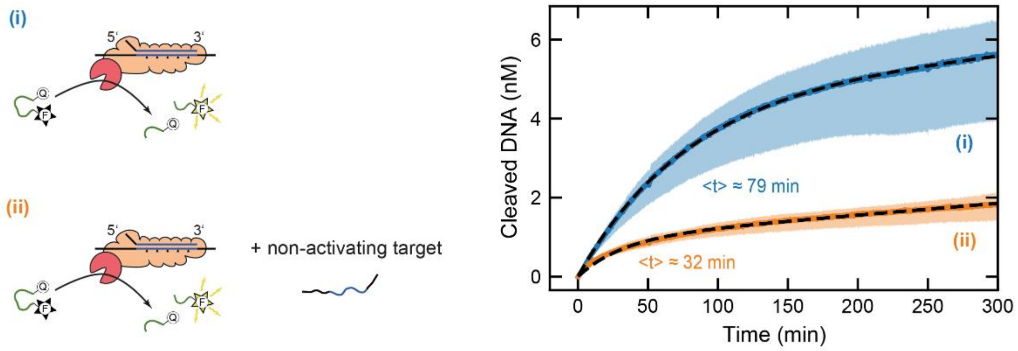

Figure S11: DNase activity in the presence of non-activating target RNA. Cleaved ssDNA as function of time measured for the Csm complex (2 nM), preincubated with activating target RNA S3/a (2 nM) and ssDNA (200 nM). The reaction was initiated by adding $MnCl_2$ (0.5 mM) without (i) or with (ii) non-activating target RNA S3/n (50 nM). Solid lines represent the mean and colored areas indicate maximum and minimum values of three measurement repeats. Fits (dashed lines) of an exponential with an additional background rate (Equation (5) main text) to the experimental data provided a mean reaction time in the absence of non-activating target RNA of (79 ± 1) min and in its presence of (32 ± 1) min.

Table S1: RNA substrate sequences used in this study.

Identity	Sequence 5'-> 3'
crRNA<br>(present in WT and RNase dead mutant)	ACGGAAACUUUCGUAACUGUUUAAUUCUGUUCACUUAUUC
activating target RNA S3/a	GGGAAACGACGGCCAGUGCCAAGCUUGCAUGCCUGCAGGUCGAC<br>UCUAGAGGAUCCCCAAAUAUAAGGUG**GAAUAAGUGAACAGAAUU**<br>**AAACAGUUACGAAA**AAAAAAAAGGGUACCGAGCUCGAAUUCGUA<br>AUCAUGGUCAUAGCUGUUUCCUGUGUGAAAUUGUUAUCCGCUCA<br>CAAU
non-activating target RNA S3/n	GGGAAACGACGGCCAGUGCCAAGCUUGCAUGCCUGCAGGUCGAC<br>UCUAGAGGAUCCCCAAAUAUAAGGUG**GAAUAAGUGAACAGAAUU**<br>**AAACAGUUACGAAA**GUUUCCGUGGGUACCGAGCUCGAAUUCGUA<br>AUCAUGGUCAUAGCUGUUUCCUGUGUGAAAUUGUUAUCCGCUCA<br>CAAU
non-specific RNA NS	GGGAAACGACGGCCAGUGCCAAGCUUGCAUGCCUGCAGGUCGAC<br>UCUAGAGGAUCCCCAAAUAUAAUUUC**GUAACUGUUUAAUUCUGU**<br>**UCACUUAUUCCACC**AAAAAAAAGGGUACCGAGCUCGAAUUCGUA<br>AUCAUGGUCAUAGCUGUUUCCUGUGUGAAAUUGUUAUCCGCUCA<br>CAAU
5' mismatched (9nt) target RNA 5'mm	GGGAAACGACGGCCAGUGCCAAGCUUGCAUGCCUGCAGGUCGAC<br>UCUAGAGGAUCCCCAAAUAUAAGGUG**GAAUAAGUGAACAGAAUU**<br>**AAACACAAUGCUUU**AAAAAAAAGGGUACCGAGCUCGAAUUCGUA<br>AUCAUGGUCAUAGCUGUUUCCUGUGUGAAAUUGUUAUCCGCUCA<br>CAAU
3' mismatched (9nt) target RNA 3'mm	GGGAAACGACGGCCAGUGCCAAGCUUGCAUGCCUGCAGGUCGAC<br>UCUAGAGGAUCCCCAAAUAUAACCAC**CUUAUUCACAACAGAAUU**<br>**AAACAGUUACGAAA**AAAAAAAAGGGUACCGAGCUCGAAUUCGUA<br>AUCAUGGUCAUAGCUGUUUCCUGUGUGAAAUUGUUAUCCGCUCA<br>CAAU
5' truncated target RNA 5'trunc	GGGAAACGACGGCCAGUGCCAAGCUUGCAUGCCUGCAGGUCGAC<br>UCUAGAGGAUCCCCAAAUAUAAGGUG**GAAUAAGUGAACAG**
3' truncated target RNA 3'trunc	GG**UUAAACAGUUACGAAA**AAAAAAAAGGGUACCGAGCUCGAAUU<br>CGUAAUCAUGGUCAUAGCUGUUUCCUGUGUGAAAUUGUUAUCCG<br>CUCACAAU
Internally labeled activating target RNA S3/iL	GGGAUCCCCAAAUAUAAGGUG**GAAUAAGUGAACAGAAU**[U-<br>ATTO647N]**AAACAGUUACGAAA**AAAAAAAAGGGUACC

Table S2: DNA oligonucleotide sequences used in this study.

Identity	Sequence 5'-> 3'
Green FCS probe<br>(complementary to 5'-end of RNA substrates)	ATGCAAGCTTGGCACTGGCCGTCGTTTCCC-**[ATTO532]**
Red FCS probe<br>(complementary to 3'-end of RNA substrates)	**[ATTO647N]-**ATTGTGAGCGGATAACAATTTCACACAGGA
Bulk probe	**[6FAM]**-TTAGCT-**[BHQ1]**
unlabeled ssDNA	AGCGATAGAAGACAGAC
TIRF probe<br>(complementary to 3'-end of RNA substrates)	[**AlexaFluor647**]-<br>ATTGTGAGCGGATAACAATTTCACACAGGA-<br>[**AlexaFluor647**]
TIRF anchor<br>(complementary to 5'-end of RNA substrates)	ATGCAAGCTTGGCACTGGCCGTCGTTTCCC-[**Biotin**]

REFERENCES

1. Lakowicz, J.R. (2006) Principles of fluorescence spectroscopy (3rd ed). Springer, New York, Berlin.
